# “It gives me confidence and value”: evaluating All_Ears@UoS patient and public involvement and engagement (PPIE) group three years on

**DOI:** 10.1186/s40900-026-00986-2

**Published:** 2026-10-08

**Authors:** Kate Hough, Sarah Smith, Roger Byatt, Marcia Hudson, Tracey Newman

**Affiliations:** 1 Clinical and Experimental Sciences, Faculty of Medicine, University of Southampton, Southampton, UK; 2 Auditory Implant Service, University of Southampton, Southampton, UK; 3 ALL_EARS@UoS PPIE Group, University of Southampton, Southampton, UK

**Keywords:** Patient and public involvement, Evaluation, Hearing loss, Audiology, Standards, Engagement, Cochlear implant, Impact, Inclusion

## Abstract

**Background:**

All_Ears@UoS is a thriving patient and public involvement and engagement (PPIE) group that was established in March 2022. The ethos of All_Ears is to create an enduring partnership between researchers and people with lived experience of hearing loss to shape and inform research. This paper describes the findings from an evaluation to measure the perceived impact, value and engagement of the group. The UK Standards for Public Involvement were used as a framework. These include; Communications, Governance, Impact, Working together, Support and learning and Inclusive opportunities.

**Methods:**

Two surveys were co-designed with members of All_Ears and shared via an online platform to evaluate what was working well and what could be improved from the group members’ and researchers’ perspectives. Each survey combined demographic questions, rating scale questions to measure satisfaction and open-text questions to gather feedback. Descriptive statistics were used to describe the satisfaction and demographic data. Reflexive thematic analysis was used to analyse the free-text responses.

**Results:**

Strengths aligning to several of the UK Standards for Public Involvement were identified. Thematic analysis of group member responses developed six themes, encompassing the perceived value of the group for research, opportunities for learning and raising awareness, the benefits of the group, aspects that were working well or less well, and considerations around diversity and inclusivity. Four themes were developed from the analysis of researcher responses. Three themes reflected strong support for PPIE within research, while one focused on strengths and challenges in how the group was organised and run. Suggested areas for improvement were to enhance training opportunities to develop skills to support involvement and greater flexibility around meeting scheduling. Group members suggested that a faster reimbursement process is necessary while researchers highlighted a need for increased visibility of public involvement within the University.

**Conclusions:**

This evaluation has highlighted the perceived value, impact and benefits of embedding enduring PPIE on research and at an individual and societal level from the group members’ and researchers’ perspectives. We hope the findings enable biomedical and clinical researchers to see the value of building partnerships with patients and the public.

**Clinical trial number:**

Not applicable.

**Supplementary Information:**

The online version contains supplementary material available at https://doi.org/10.1186/s40900-026-00986-2.

## Abstract (plain language summary)

Patient and public involvement and engagement (PPIE) is researchers working in partnership with people with lived experience to shape and guide research. In March 2022, we set up a PPIE group called All_Ears@UoS for people with lived experience of hearing loss and deafness. Our aim was to build a partnership between people with lived experience of hearing loss and researchers. All_Ears@UoS discusses, informs and influences hearing research. Three years after the group launched, we carried out an evaluation to measure the perceived impact, value and engagement of the group. We used surveys to ask and measure what was working well and what could be improved about the group for group members and researchers. Group members are members of the public with lived experience of deafness. Researchers are people who have discussed their research with the group members. Group members identified that communication and the diversity and inclusivity of the group were strengths. Both group members and researchers reported they felt that the group had a positive impact on themselves, the deaf community, wider society, and the research. Group members felt that the reimbursement process, flexibility of meetings, and more training opportunities were things that could be improved. For researchers, increased visibility at the University, more training opportunities and changes to meeting scheduling were areas to be improved. We report on how we will use these findings to further develop the group and to address the issue where improvements are needed.

**Supplementary Information:**

The online version contains supplementary material available at https://doi.org/10.1186/s40900-026-00986-2.

## Background

Patient and public involvement and engagement (PPIE) is increasingly recognised as integral to health and medical research [1]. Effective PPIE should enable the public and researchers who engage with it, to see that their efforts are achieving the desired outcome. Here, we evaluate the perceived impact, value and engagement of an established PPIE group for people living with hearing loss, All_Ears@UoS, from the perspectives of both group members and researchers.

Public involvement is a sharing of knowledge, or partnership, between researchers and the public that supports two-way learning [2]. Researchers acquire professional or research knowledge within their careers. The public develop experiential knowledge [3] gleaned through caregiving and or living with a condition. Public involvement explores not just a person’s lived experience but also the impact of their lived experience on their daily life. Effective partnership working is fundamental to ensuring that research is impactful, relevant and translates to improved practice [1, 4]. Partnership encourages meaningful participation and engagement. Key to this is ensuring that people with a diversity of knowledge and backgrounds can be found and brought together and that barriers to partnership working are uncovered, explored and worked through to enable trust and common purpose to be built. Community engagement can ensure the voice of diverse communities is at the core of public involvement activities. Both public involvement and community engagement are methods which acknowledge the impact of social and cultural differences.

People who are D/deaf or hard of hearing are an underserved community in research. That is, they are part of a group that are not adequately represented, included in or served by research [5]. In addition to this, the lived experience of hearing loss and deafness is largely underappreciated in society [6], despite around 18 million people in the UK living with at least some measurable hearing loss or tinnitus. The experiences, needs and communication methods of people who are deaf or hard of hearing vary depending on the degree of hearing loss or deafness. People who identify as deaf (lowercase ‘d’) often have severe to profound hearing loss, people who identify as Deaf (uppercase ‘D’) often identify as part of the Deaf community, and people who are hard of hearing often have mild to moderate hearing loss. However, how a person identifies with their deafness, or hearing loss varies and is unique to them. Over half the population aged 55 or older have hearing loss [7, 8]. While some people have marked hearing loss, or are deaf from birth, many live with a progressive, and often unpredictable, hearing loss. A range of factors impact daily living with hearing loss including; the severity of hearing loss, age and presence of common comorbidities, along with socioeconomic status, access to services and care and support networks. The daily challenges of living with hearing loss can be lessened through the use of hearing aids, cochlear implants or learning and using Sign Language and lip reading. Better understanding of the complexity and diversity of living with hearing loss through taking the time to listen to lived experience is critical, to designing more effective audiology research, and to improve health research and services [9, 10].

For underserved communities, there are multiple barriers that prevent individuals getting involved in research. These include a lack of awareness of opportunities through poor recruitment strategies, practical and financial constraints, physical disability, cultural differences and a lack of trust in health research. Poor awareness of the accessibility and communication needs for deaf and hard of hearing people by the very agencies tasked to make research and healthcare provision effective can make these barriers greater [9, 10]. This lack of awareness can limit or preclude access to information and services. Researchers must make a deliberate effort to reduce the barriers that exist for different communities to generate the evidence needed to develop and inform healthcare improvements.

**Fig. 1:**
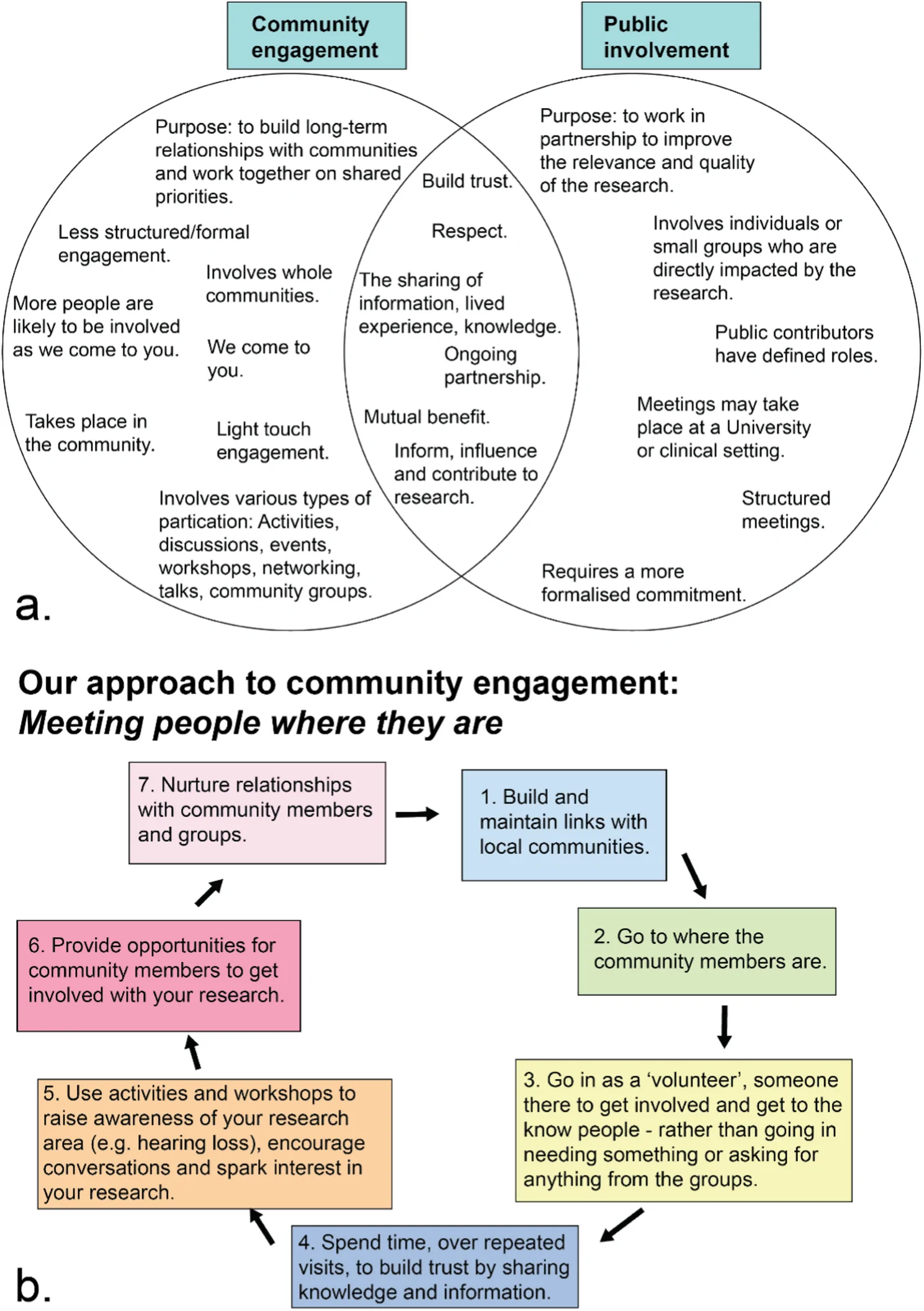
The intersection between community engagement and public involvement. **a**. Schematic outlining the similarities and differences of community engagement and public involvement. **b**. The seven steps to our community engagement approach - Meeting people where they are

All_Ears@UoS, a PPIE group of people with lived experience of hearing loss and deafness [11], was established in March 2022. Our aim was to build an enduring group where people with lived experience worked in partnership with researchers to improve hearing research and outcomes for people with hearing loss. Since its inception, we have co-developed and expanded the reach of the group. The group now has 60 + diverse members, aged between 20 and over 80-years-old. We have used community engagement to build trust with people from diverse backgrounds. Through this, we have shared information and knowledge and encouraged people to get involved with research. Community engagement is distinct from public involvement as it aims to build long-term relationships with communities and work together on shared priorities. Public involvement aims to work in partnership to improve the relevance and quality of the research (Fig. 1a). Over several years, we have been going into the local community to raise awareness of hearing and brain health. Through this, we have learnt how to build trust and relationships with underserved groups, and we have developed a seven-step approach to community engagement. This is described as ‘Meeting people where they are’ (Fig. 1b). We have raised awareness of hearing and cochlear implants, and we have encouraged people to join All_Ears. Nearly half of our members have been recruited through community engagement. This has increased the diversity of the group over time.

The public members involved with All_Ears and the breadth of researchers who engage with group members has changed over time. All_Ears has become more representative of the deaf and hard of hearing community and of the community in the Hampshire and Solent region. This evaluation aims to explore the impact of these changes over time.

Despite considerable changes to the make-up of the group, many aspects have remained constant including our aims and ethos. We make a deliberate effort to highlight the value of social, cultural and demographic differences among group members and researchers. We work alongside group members and researchers to co-run public engagement activities and exhibits at community events and science festivals. The format of the in-person meetings has not changed: they are a combination of small-group activities and topic-led discussions. Group members and researchers work together on activities designed to create the time and space to share personal experiences. The topic-led discussions are led by researchers wanting to explore research ideas with All_Ears members. These discussions can spark new ideas and highlight issues that are important to our group members that could be explored and developed into research projects. Researcher-led discussions have spanned early ideas that might lead to projects through to discussions of projects under development and reporting on progress with research projects that are underway.

An evaluation of the first year of All_Ears@UoS highlighted both the value of the group to members and the challenges of our PPIE activities [11]. The paper details how the group was established, co-developed and its effectiveness from the perspective of the group members [11]. Group members were clear that they found personal value through the group and described feeling listened to and empowered. They felt the group were adding value to wider society through the collaborative efforts to raise awareness of hearing loss and cochlear implants. However, they also identified that the time and format of meetings prevented some members from participating. Alongside the feedback from group members, we reflected on the challenges that we faced in supporting and enabling diversity and inclusion within the group. We also considered the intersection of researchers with the group. The findings informed our approach for the next phase of the group which focused on how to grow the group and develop effective ways for researchers to contribute and be informed by the group.

Regular review of PPIE activities using a robust evaluation framework can support effective PPIE and help with the sharing of best practice with researchers and public involvement professionals. Despite the increasing number of tools and frameworks available to evaluate PPIE activities, the quality of evaluation of PPIE activity is often insufficient and inconsistent [12, 13]. This limits the quality of evidence used to inform assessment of the wider impact and value of PPIE. Building on our first evaluation of the group in 2022 [11], we have used the UK Standards for Public Involvement as a framework for this evaluation [14]. The standards are a description of what good public involvement looks like and cover six important ‘values based’ areas including communication, governance, impact, working together, support and learning, and inclusive opportunities [14]. Other groups have used these standards as a framework for the evaluation of their public involvement activities [15–17].

Three years into All_Ears, we wanted to use the UK Standards for Public Involvement to evaluate the perceived impact, value and engagement of the group from the group members’ and the researchers’ perspective. This was important to better understand the effectiveness of the partnership between group members and researchers and to determine where further improvements should be made. The learnings from this evaluation may be applicable to researchers setting up and running similar programmes.

## Aims

To evaluate the All_Ears PPIE group, three years on, to determine the perceived impact, value and engagement of the group by;

1. Monitoring the activities, engagement, outputs of the PPIE group over time.
2. Using the six UK Standards for Public Involvement as a framework, to determine what is working and what can be improved from both the group members’ and researchers’ perspective.

## Methods

### Monitoring of PPIE activities

Data have been captured over time to record the activities, meetings, engagements and outputs from the group. The demographic characteristics of the group including how members were recruited has been captured.

### Evaluation of the group using anonymous surveys

#### Survey design

Two co-designed surveys to evaluate the perceived impact, value and engagement of the group were developed. The group members’ survey aimed to gather the perspective of All_Ears group members. The second, the researchers’ survey, was designed to gather the perspective of researchers who have worked with All_Ears.

An evaluation steering group of three self-selected members of All_Ears, and PPIE co-leads was established. The steering group developed a way of working, discussed the process and content of the evaluation and designed and refined the surveys. The first evaluation of the group in 2023 [11] was used as a template. A development in this evaluation was a discussion about the need, value and sensitivities of collecting and reporting the demographic information of respondents. A discussion with the wider All_Ears membership about the format and content of the surveys, and whether demographic information should be collected, was held at a PPIE meeting. There was near consensus that suggested demographic data should be collected. Feedback from the wider group was used to refine the surveys which were then reviewed by the evaluation steering group. The feedback and points raised through the review were addressed and the documents were submitted for independent ethical review and approval through the University ethics committee.

#### Survey structure

Both group member and researcher surveys included a combination of (i) Questions using a rating scale (strongly agree, agree, neither agree nor disagree, disagree, strongly disagree or rate 0–5), (ii) Short and longer open-text questions and (iii) Demographic self-identification using a check box list. The UK Standards for Public Involvement were used as a framework for the rating scale questions and open-text questions [14]. Participants were asked to indicate their agreement with a series of statements which aligned with each of the six standards. Open-text questions were included to give participants the opportunity to share their wider perspectives to enable us to gather more detailed, qualitative data. See additional file 1 for the survey drafts (group members 1a, researchers 1b). The questions, written in plain English, were: [1] What has worked well [2]? What areas would you like to see improvements in [3]? What do you see as the impact of being involved within this group [4]? Describe any benefits of being part of All_Ears.

Group member respondents were asked their (i) age, (ii) ethnicity, (iii) gender, (iv) current employment status, (v) sector of employment, (vi) first part of postcode. In the researchers’ survey, researchers were asked to identify the institution they were based at and their current research role e.g., doctoral researcher, clinical researcher, principal investigator, etc.

Any group members who were in the group for the first evaluation in May 2023 were asked to reflect on their experiences since that evaluation. This was our first evaluation of the researchers’ perspective, so the survey asked them to consider their experiences since their first involvement with the group.

#### Ethics

Ethical approval for the evaluation was granted by University of Southampton Ethics Committee, reference number ERGO/103,288.

#### Consent

Group members and researchers were asked to read the combined participant information sheet and consent form (https://acrobat.adobe.com/id/urn:aaid:sc:EU:1e3067ef-35c1-497a-b22a-21a20d685d74) before completing the survey. Participants were asked to confirm that they were over 18 and that they agreed to participate in the evaluation by completing the survey. They were asked to give their consent for the use of the fully anonymised data in peer-reviewed and grey publications, and for sharing of the findings with other clinical centres and through internal university channels.

### Survey administration

The surveys were made available to the group members and researchers using Microsoft Forms and were live for four weeks in June 2025.

### Analysis of data

Researcher (KH) completed an initial review of the raw data before sharing with the evaluation steering group members for their initial comments and interpretation. Descriptive statistics were used to analyse the demographic data and rating scale questions. When evaluating changes in attitudes between the first and second evaluation, the analyses were primarily descriptive due to small sample sizes and independent respondent groups.

The answers to the open-text questions on the surveys were analysed using Braun and Clarke’s six phase process of reflexive thematic analysis [18–20] by researcher and group co-lead (KH). The same approach was taken for the thematic analysis as described in our first evaluation [11]. The six phases include (1) Data familiarisation and writing familiarisation notes, (2) Systematic data coding, (3) Generating initial themes from coded and collated data, (4) Reviewing and developing themes, (5) Refining, defining and naming themes, (6) Writing up [21]. The analysis was managed using the standard software NVIVO. Inductive coding was used whereby there were no pre-determined codes. The initial review of the data was completed by reading and extracting the quotes or phrases (phase one). A set of initial codes were developed based on the review (reading and rereading) of the data (phase two). From these initial codes, initial themes were developed (phase three). In phase four, the themes were reviewed to determine quality of the theme and whether there was enough meaningful data to support that theme. In phase five, the themes were defined to determine the story of the analysis then named. The final phase was writing up the analysis and drawing conclusions from the themes.

KH, who carried out the reflexive thematic analysis, is a researcher and has been a co-lead of All_Ears from the beginning. Over time, KH has built interpersonal relationships with group members and is deeply invested in the success of the group. In addition, KH carried out the thematic analysis for the first evaluation [11]. These factors likely influenced how the data were interpreted during the thematic analysis. Throughout the analysis, KH examined and reflected upon this by recording reflexive notes and discussing this with the evaluation steering group.

The survey findings were discussed with group members in a meeting in October 2025. In the meeting, group members were asked to discuss, comment on and provide actions against the areas that were described in the survey as working well and areas identified as needing improvement.

### Reporting

Guidance for Reporting Involvement of Patients and the Public (GRIPP2) [22] long form has been used to format the reporting of these findings (additional file 2).

## Results

### Findings from ongoing monitoring of PPIE activities

A summary of the various involvement and engagement activities that group members have been involved with to date is shown in Table 1. Group members have supported research in a variety of ways, from contributing to meetings, reviewing project documents and grant applications and supporting presentations and public engagement events. Additional file 3 shows the breakdown of number of attendees for each meeting, including whether the attendees were new and where the attendees were recruited from.

**Table 1:** Summary of involvement and engagement activities that group members have been involved with

Involvement and engagement activities	Number
In-person meetings	22
Online meetings	2
Review project/study documents	17
Involvement in grant applications	8
Ethics application panel	3
Public engagement events	11
Co-produced presentations	2

### Findings from group members’ survey

#### Demographic characteristics of survey respondents

The response rate of the group members’ survey was 40% (18/44). All respondents were over 30 years old, and the majority (44%, 8/18) were aged between 50 and 69 years old. The data showing age, gender and ethnicity of the respondents [Table 2] enable comparison with the demographic of respondents in our evaluation in 2023. A high proportion (94%, 17/18) of the 2025 group member respondents were female, in contrast to 64% (7/11) in the 2023 evaluation. This reflects that at the time of the 2025 survey, 75% of the public membership identified as female (data not shown). The ethnic diversity of the survey respondents was greater in 2025 compared to the 2023 with 61% (11/18) White British, 6% Asian or Asian British (1/18) and 33% Black, Black British, Caribbean or African (6/18). The employment status of the group members, [Table 3] shows the majority described themselves as ‘Retired - with several community commitments’, with 22% (4/18) of members describing themselves as being employed either full or part time.

**Table 2:** Comparison of the demographic characteristics of group member respondents between the first and second evaluation

Age	All_Ears survey respondents (2025)	Percentage of All_Ears survey respondents (2025)	All_Ears survey respondents (2023)	Percentage of All_Ears survey respondents (2023)
	Number	% (*n* = 18)	Number	% (*n* = 11)
18–29	0	0	0	0
30–49	3	17	1	9
50–69	8	44	4	36
70–79	5	28	4	36
80 and above	2	11	2	18
**Gender**				
Female	17	94	7	64
Male	1	6	4	36
**Ethnicity**				
White British	11	61	10	91
Asian or Asian British	1	6	0	0
Black, Black British, Caribbean or African	6	33	1	9
Mixed or multiple ethnic groups	0	0	0	0
Other ethnic group	0	0	0	0

**Table 3:** Employment status of group member survey respondents

Employment	
Employed, full time	2
Employed, part-time	2
Full-time carer	1
Not employed, looking for work	0
Not employed, NOT looking for work	0
Retired – with several community commitments e.g. All_Ears, member of school or other community board	12
Retired – community commitment All_Ears	2
Disabled, not able to work	0

Note. Respondents could select more than one response if appropriate

Outward postcodes were collected to determine the area that members were living in. All postcodes in the dataset related to specific geographical locations, with no non-geographic codes returned. The majority 56% (10/18) of respondents have a Southampton (SO) postcode, covering 8/23 different SO districts. Those living in Southampton live within a 15-mile radius of the Highfield campus of the University. The remaining group members live in areas across Hampshire, Portsmouth, Basingstoke and Dorset. Some members travel up to 40 miles each way to attend meetings and events. A map [additional file 4] identifies the location of the in-person meetings, which are held at the Highfield campus of the University of Southampton, and the distance in miles to surrounding regions where group members travel from.

The survey data demonstrates All_Ears is engaging with a broad demographic. Of 317 local authorities in England, Southampton is ranked 55th most deprived. The Index of Multiple of Deprivation (IMD), a measure of socioeconomic deprivation by area, was estimated using outward postcode data and indicated the inclusion of a broad demographic spread across affluent (IMD 7–9) and more deprived (IMD 1–3) areas.

### Rating scale questions

#### Summary of rating scale questions

**Fig. 2:**
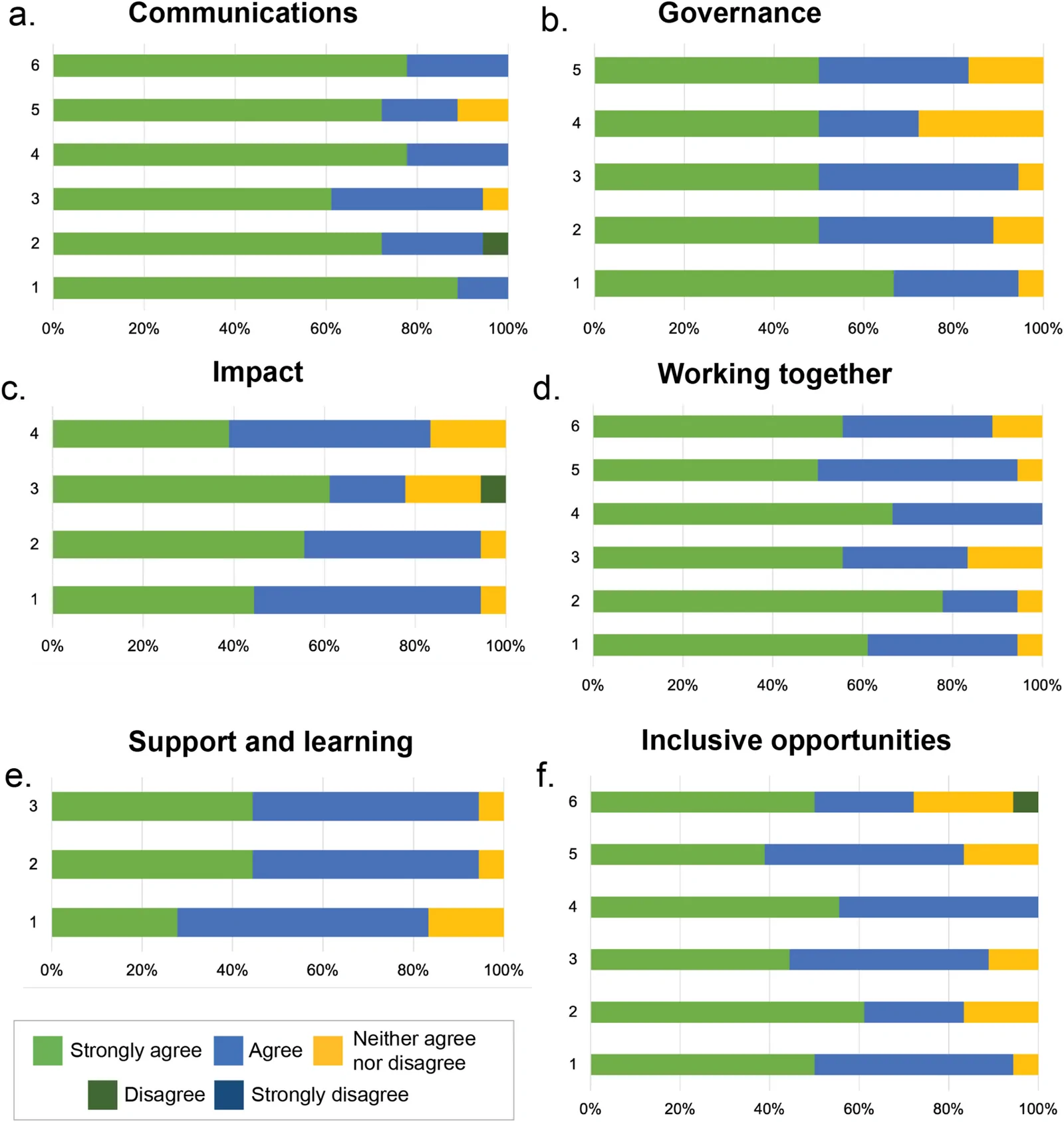
Stacked bar charts showing the rating scale question scores (%) from the group members’ survey. Statements (y-axis) which aligned with each of the six UK Standards for Public Involvement were included (30 statements in total). The numbers beside the bar summarise each statement within the standard and match the numbers listed next to the statements written in full in Table 4 and additional file 5a and 5b

The group member survey asked participants to rate 30 statements. The statements were descriptions of effective public involvement activities or ethos. Table 4 displays the statements which align with each of the UK Standards for Public Involvement. The number next to each statement in the table corresponds to the numbers in Fig. 2. A higher proportion of positive responses (strongly agree/agree) would suggest respondents felt the group’s PPIE activities aligned closely with the six UK Standards for Public Involvement. The summary of the ratings (%) of the statements (y-axis) for each of the standards is in Fig. 2. Additional files 5a and 5b show the detailed scores alongside each of the statements (the number beside the statement links to the numbers seen in Fig. 2).

**Table 4:** Statements that were used for the rating scale questions in the group members’ survey

UK Standards for Public Involvement						
Communications	There are clear and informative communications about upcoming meetings, written work, and activities (1).	The methods for communicating in meetings and via emails are inclusive for the whole group (2).	The methods for communicating in meetings and via emails are flexible for the whole group (3).	There are regular opportunities to offer feedback about meetings, project ideas and activities (4).	My feedback is gathered, acted on and shared back to the group (5).	Our activities, learning and achievements (positive and negative) are shared amongst and the group and more widely (6).
Governance	Group member’s voices are heard, valued, and respected in decision making (1).	Public involvement plans are in place, and these plans are regularly monitored, reviewed, and reported on (2).	Throughout the organisation, there is visible and accountable responsibility for public involvement (3).	Realistic resources such as money, staff, time are allocated for public involvement (4).	The privacy of personal information is protected by collecting and using it in a suitable way (5).	
Impact	Group members are involved in deciding how the impact of their involvement should be assessed (1).	Information is collected to help assess impact of the involvement such as who has been involved and how they were involved (2).	Time and activities such as meetings are allocated to reflect and evaluate the patient and public involvement (3).	My involvement has an impact on research (4).		
Working together	The aims and purpose of the group have been jointly agreed and defined by the group (1).	I feel valued and able to share ideas freely without restriction (2).	The practical arrangements, preferences, and requirements for working together have been discussed, addressed, and agreed as a group (3).	In activities, the team are clear about objectives, how to achieve them and how to present the results (4).	There is a shared understanding of roles, responsibilities, and expectations for patient and public involvement (5).	Our individual ideas and contributions are recognised, addressed and shared with the group (6).
Support and learning	There are opportunities to improve one’s skill set to enable more involvement (1).	Resources have been designated to support learning and development opportunities for both researchers, ALL_EARS members, and staff (2).	There is a culture of learning by doing, building upon and sharing best practice between the whole group/team (3).			
Inclusive opportunities	Patient and public group members are involved in the research from the earliest stages (1).	Barriers to involvement such as payment or accessibility have been identified and addressed (2).	Information about activities is shared widely in ways that are accessible and appeal to different communities (3).	There is choice in the opportunities that are offered for group members to get involved with research (4).	The processes we have for getting involved result in an inclusive and diverse group (5).	The group is a true representation of the Deaf or hard of hearing community (6).

The responses were positive for 5 out of 26 statements against the standards and either positive with some ambivalence (neither agree nor disagree) for the majority of statements (22/30). Aspects of Communications, Working together and Inclusivity were identified as strengths. For 24 out of 30 statements, at least one group member selected at midpoint (neither agree nor disagree) suggesting some ambivalence.

Respondents disagreed with three statements across Communications, Impact and Inclusive opportunities. These were: ‘The methods for communicating in meetings and via emails are inclusive for the whole group [Communications]’, ‘Time and activities such as meetings are allocated to reflect and evaluate the patient and public involvement [Impact]’, ‘The group is a true representation of the Deaf or hard of hearing community [Inclusive opportunities]’. These topics will be discussed in group meetings to determine how we can make improvements. No group respondent selected strongly disagree for any of the statements. Similarly, 23/26 statements had between 5% and 25% of respondents stating they neither agree not disagreed.

### Differences in responses between the first and second evaluation

15 of the statements were rated in both the 2023 [11] and this evaluation. We evaluated the descriptive differences in how attitudes of the group members have changed since the evaluation in 2023. As the demographic profile of the group member respondents changed considerably between the first and second evaluation, changes in attitudes to the statements may not just reflect a change over time but could also reflect who the group member respondents were.

**Fig. 3:**
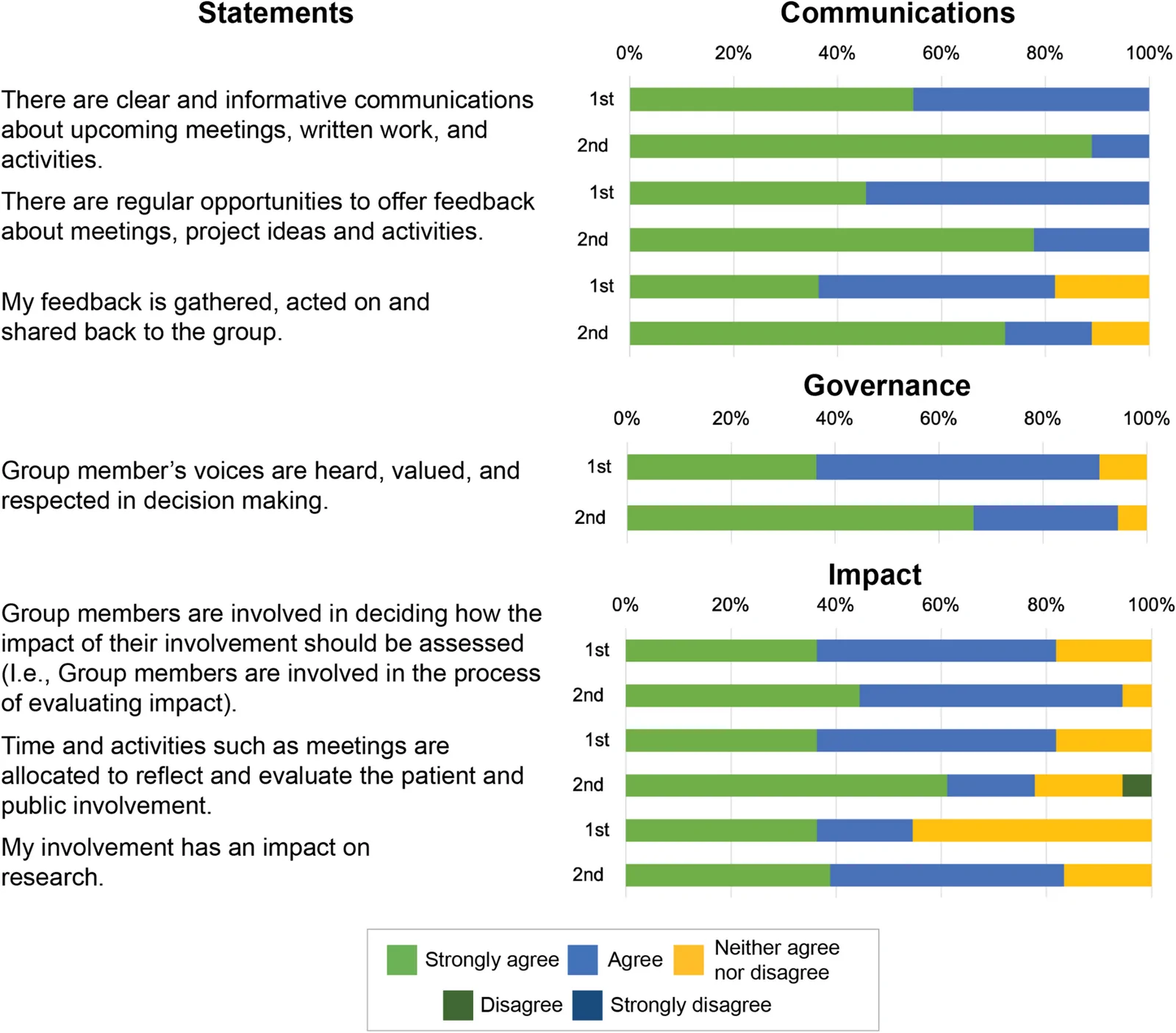
A comparison of rating scale question responses between the first and second evaluation. Stacked bar charts comparing the scores (%) from the rating scale questions for the statements from the 2023 evaluation [1st ] and this [2nd ] evaluation. The statements related to Communications, Governance and Impact

**Fig. 4:**
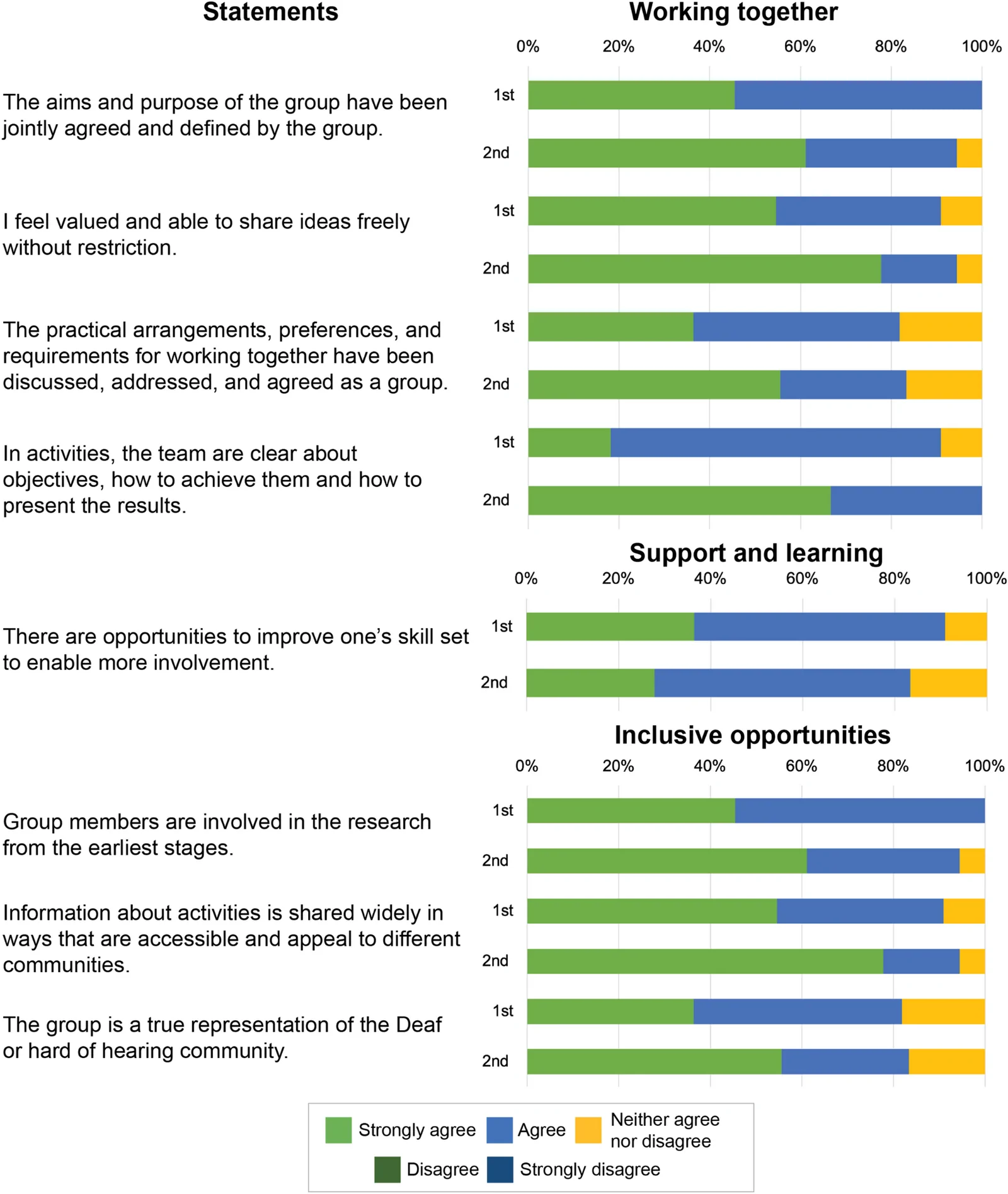
A comparison of rating scale question responses between the first and second evaluation. Stacked bar charts comparing the scores (%) from the rating scale questions for the statements from the 2023 evaluation [1st ] and this [2nd ] evaluation. The statements related to Working together, Support and learning, and Inclusive opportunities

For 14 of the 15 statements related to Communications, Governance, Impact, Working together and Inclusive opportunities, the percentage of group respondents that selected ‘Strongly agree’ increased in the second evaluation (see Figs. 3 and 4 by comparing the green bars). This shows a substantial positive change in attitudes where a higher percentage of the group member respondents more strongly agreed that our group and activities align with the key areas of public involvement. There was a decrease in the percentage of group respondents that ‘Strongly agreed’ in the second evaluation to one statement which was ‘There are opportunities to improve one’s skill set to enable more involvement’. This aligned with the standard Support and learning (Fig. 4). Overall, these findings suggest an improvement in the respondents’ attitudes across areas which relate to Communications, Governance, Impact, Working together and Inclusive opportunities. An area for improvement is to ensure group members feel there are opportunities for them to improve their skill set to enable more involvement.

### Thematic analysis of open-text questions

Six themes aligning with at least one of the six standards, summarised in Table 5, were developed from the reflexive thematic analysis of the group members’ responses to the open-text questions. The UK Standard for Public Involvement which most aligns with each subtheme is shown in Table 5. Impact and Inclusive opportunities were the two standards with the most prominent subthemes. Additional file 6 outlines the full quotations from the free-text responses which align with the themes and subthemes. Participant identifiers for group members are indicated in square brackets e.g. [G1].

**Table 5:** Themes and subthemes developed from the group members’ survey

Theme	Subtheme	UK Standard for Public Involvement that the subtheme most aligns with
1. Supporting research		Impact
2. Learning opportunities and knowledge exchange	2.1 Knowledge exchange (outward facing) 2.2 Personal learning (inward facing)	Support and learning
		Support and learning
3. Raising awareness	3.1 Raising awareness within deaf and hard of hearing community 3.2 Raising awareness and sharing knowledge with the wider community 3.3 Using public engagement activities to raise awareness	Impact
		Impact
		Impact
4. Impact, benefits and opportunities the group brings	4.1 Impact of the group 4.2 Personal benefit of being involved 4.3 Opportunities the group brings	Impact
		Impact
		Impact
5. Diversity and inclusivity within the group	5.1 Involvement with diverse communities 5.2 The group is inclusive 5.3 Lack of involvement from some communities	Inclusive opportunities
		Inclusive opportunities
		Inclusive opportunities
6. Running the group	6.1 Leadership of the group 6.2 Areas that are working well 6.3 Areas that need improvement 6.4 Suggestions to improve meetings	Communications
		Working together
		Governance
		Inclusive opportunities

### Theme 1: Supporting research

Group members reflected on the breadth of ways they have supported research that they feel aims to improve outcomes and reduce the daily challenges experienced by people who are deaf or hard of hearing. This included contributing to articles, providing feedback on written material and ethics applications and contributing to group discussions. Group members gave examples of their involvement which highlighted how they support research. Examples (verbatim in quotation marks) included ‘feedback on some proposed projects [G3]’, they ‘responded to a proposed ethics document re a piece of research [G5]’, they were involved with ‘writing materials, taking part in research [G8]’ and two members commented that they had contributed to ‘articles – BMJ, CIICA and All Ears website [G15]’. The opportunity to contribute to research through public engagement activities such as ‘Helping out at open days and New Forest Shows [G11]’, was described as a benefit. Some group members described how they felt that their involvement in the group and contribution to research was impactful, valued and used to improve the lives of those who are deaf or hard of hearing.

### Theme 2: Learning opportunities and knowledge exchange

The positive benefits and impact of sharing and receiving knowledge between researchers, group members and members of the deaf and hard of hearing community as well as the wider community were discussed.

#### Subtheme 2.1: Knowledge exchange (outward facing)

Several group members described the learning that takes place from each other, ‘The All_Ears group have learnt much from each other [G1]’, whether it be through ‘spreading tips about how to make life easier, e.g. Speech to Test apps [G11]’ or through listening. The personal benefit of learning from each other, researchers or by attending group meetings was highlighted.

#### Subtheme 2.2: Personal learning (inward facing)

The group members reflected on their personal learning, the opportunities to learn new skills and about the research process. Support and learning are one of the UK Standards for Public Involvement which states that we should offer and promote support and learning opportunities that build confidence and skills for public involvement in research.

### Theme 3: Raising awareness

#### Subtheme 3.1: Raising awareness within deaf and hard of hearing community

The group appreciate being able to share information with each other and raise awareness within the deaf and hard of hearing community.

#### Subtheme 3.2: Raising awareness and sharing knowledge with the wider community

Raising awareness in the wider community, including the group members’ local communities is important for them - ‘The most important thing is the awareness as a group we are able to share with particularly the grassroot community [G9]’. Some members come along to our meetings with the intention of receiving information which they can use for ‘educating the community [G16]’ or to ‘share experiences with the community [G13]’.

#### Subtheme 3.3: Using public engagement activities to raise awareness

Public engagement activities were highlighted as way of raising awareness. Group members described taking part in ‘open days [G7]’ and public engagement ‘activities to raise awareness of the effects of deafness to general public [G13]’. All_Ears group members have now taken part in 11 public engagement activities. At the events, when we talk to members of the public about cochlear implants and how they work, having people with lived experience of cochlear implants there to show and tell – brings our exhibits to life.

Suggestions were made about how to improve public engagement activities. At events, one member described it being important to have ‘listening breaks for all on the stand at Open Days as prolonged exposure to noise is harmful [G8]’. A further suggestion was to ensure more ways to ‘record interactions [G8]’ with the public.

### Theme 4: Impact, benefits and opportunities the group brings

The perceived impact of the group to the individual, community and people’s lives were discussed. A need for further work to measure and demonstrate the impact of the group was highlighted. The UK Standard for Public Involvement for Impact states it is important to: Seek improvement by identifying and sharing the difference that public involvement makes to research. Before the evaluation survey went live, a group meeting was dedicated to a discussion about what was meant by impact in public involvement. The definition of impact as ‘Impact refers to the difference or change that happens because of something we do. Impact means the positive changes or benefits that come from your involvement’ was used. This definition and examples of actions that could lead to impact in PPIE were discussed. Group members were asked to think about the impacts of their involvement in the group between March 2023–2025 using the ripple effects mapping exercise [23].

#### Subtheme 4.1: Impact of the group

Impacts of being involved have been described as ‘better awareness of deaf challenges [G16]’ and ‘greater awareness of the impact of deafness on the individual which means I now communicate differently. I’m more aware of how deafness can affect the lives of all involved not just the person who is hard of hearing [G14]’. Two group members described the impact of their contributions as being clear. Whereas one member felt unsure if the investment in the group had led to an impact. One group member indicated that they were interested to see ‘how All_Ears and our participation impacts hearing loss and cochlear implants across the Nation/local environment [G1]’.

#### Subtheme 4.2: Personal benefit of being involved

Personal benefits of being involved with the group such as increased confidence were reflected upon. For public involvement to be effective and enduring, it is essential that PPIE co-leads and researchers take the time to listen, get to know members and to always provide feedback on contributions they have received from group members. Five group members highlighted feeling valued as a benefit of being involved with the group. Meeting new people, ‘getting to know each other [G17]’ and having the opportunity to keep informed about latest research and information were described as personal benefits.

#### Subtheme 4.3: Opportunities the group brings

Being part of the group provides different opportunities for group members to get involved, such as ‘a diverse range of projects [G15]’, opportunities to ‘give(ing) something back [G18]’ and ‘a chance to eventually have an impact on the lives of people like me [G5]’. One benefit of being part of All_Ears was the opportunity to benefit the deaf and hard of hearing community. This is a strong value of the group, as described in theme 3.

### Theme 5: Diversity and inclusivity within the group

Positive experiences of diversity and inclusivity within the group were reflected upon.

#### Subtheme 5.1: Involvement with diverse communities

All_Ears is committed to involving people from a breadth of communities. This has been reflected in responses by group members when asked to discuss what has worked well – ‘the group is diverse [G2]’ and that we are ‘engaging everyone from various backgrounds [G3]’. There is ‘encouragement of a wide range of participants with lived experience of varying degrees of hearing loss [G6]’ and ‘integration and inclusion of the multicultural diverse community [G9]’. When given the opportunity to clarify their answers for rating scale questions related to ‘*Inclusive opportunities’*, one group member commented ‘On the grounds that there is always room for improvement there could be more diversity but any lack is due more to people not coming forward rather than lack of opportunity to do so. The All_Ears organisers try hard and in inventive ways to approach as wide a range of age, ethnicity, gender, and type of hearing loss as possible [G8]’.

#### Subtheme 5.2: The group is inclusive

The group was described as inclusive and a space where group members felt that they were ‘all treated equally [G2]’ and that meetings were ‘very inclusive and accessible’ and ‘I never feel out of place [G2]’. Nine comments were coded for this subtheme highlighting that multiple group members shared this view. The accessibility of group meetings was raised. There is ‘attention to accessibility in the group meeting [G5]’. One group member commented on the fact we only hold in-person meetings and questioned how inclusive this was. Until December 2025, all meetings had been in-person. We wanted to build meaningful and long-standing relationships with group members and felt in-person meetings would better facilitate this. The group has gained reach over time, and we are now recruiting members who live outside of the local areas. To support this and following the feedback from this evaluation, we have started running online, evening meetings. Going forwards, we aim to run a combination of in-person and online meetings to give the opportunity for different members to get involved.

#### Subtheme 5.3: Lack of involvement from some communities

A lack of involvement from the Deaf community and young people was highlighted. However, one group member described ‘being optimistic about the commitment of the group for teens [G17]’. Regarding reaching young people in their 20s, through raising awareness of All_Ears during Audiology teaching sessions led by a group co-lead (KH), university students have recently joined the group. Holding some of our group meetings online in the evening provides the opportunity for those who are studying and/or working or who have caring responsibilities to get involved.

### Theme 6: Running the group

The final theme describes attitudes towards the running and organisation of the group.

#### Subtheme 6.1: Leadership of the group

The effective leadership of the group was described as being beneficial for the successful running of the group and the enthusiasm of the research team was highlighted ‘The university team is very enthusiastic in what they do, and this really captures our attention [G2]’. Meaningful involvement from the researchers was identified and described in ‘directive from senior academics for meaningful PPI and a dedicated researcher working with the representatives [G12]’. One response suggested a need to diversify the PPIE co-lead in case the person becomes unavailable. From the start of All_Ears, the PPIE team felt it was important to have one key contact for group members to communicate with between and during meetings and to build trust and a rapport with over time. This has worked well, however going forwards, as the group builds, we need to build capacity and develop a strategy to involve other researchers in running the group.

Effective, regular communication between the PPIE co-leads and the group was described in multiple responses that described what is working well. This aligns with the UK Standard for Public Involvement - Communications, which states ‘*Use plain language for well-timed and relevant communications*,* as part of involvement plans and activities’*. There is ‘regular written communication, before, during and after every meeting which is clear to all [G6]’ and ‘we are well informed in advance of the coming meetings and events’ [G2]’. Some members mentioned that ‘email communication for purposes of sharing info, arrangements [G5]’ were working well and that ‘email updates were very helpful [16]’. Whereas two members commented that not having access to email could be a barrier to involvement, but that communication in the group is still effective.

#### Subtheme 6.2: Areas that are working well

Multiple responses referred to the friendly and welcoming environment, where there is ‘mutual support [G11]’. Our intention has been to create a welcoming environment where people feel comfortable to share their experiences and ideas, and work together. One group member shared that ‘Being part of a friendly, like-minded group of people with shared lived experiences [G18]’ was a benefit of being part of the group and another commented on the ‘friendly, helpful group [G4]’. Two responses highlighted the website as being ‘great [G18]’ and a ‘repository of useful information [G11]’. When asked to comment on areas that could be improved, three group members stated that everything was ‘working really well [G12]’. The opportunity to interact with each other in-person, in a ‘relaxed setting where people can interact both sat round table formally and less formal too in coffee break too [G7]’ was described as something that is working well.

#### Subtheme 6.3: Areas that need improvement

The reimbursement process was highlighted as an area for improvement as the unpredictable, and sometimes slow, expense process resulted in group members feeling PPIE is undervalued by the university. Institutional processes resulting in delayed compensation has been described in the literature [24]. When discussed in a recent meeting, group members commented that four weeks would be a reasonable amount of time to be reimbursed. Whereas currently, they suggested the average time was eight weeks. Another area for improvement was related to technology, specifically access to email, which could be a barrier to involvement.

#### Subtheme 6.4: Suggestions to improve meetings

Suggestions to improve meetings included one member highlighting that ‘more flexibility in the times of meetings’ would be an improvement as ‘if employed and working the time of the meetings make it difficult to participate as much as one would like [G3]’. Another member suggested that ‘maybe online meetings.’ would be beneficial for ‘wider reach [G18]’. One member suggested that having more discussion time in meetings would improve meetings.

### Findings from the researchers’ survey

#### Demographic characteristics of survey respondents

A summary of the demographic characteristics and roles of the eight researcher respondents [data in Additional file 7] shows the majority of the respondents were female (88%, 7/8) and mostly affiliated with the University of Southampton (7/8, 88%), one respondent was affiliated with the University of Cambridge. The respondents cut across disciplinary backgrounds with representation from Social Sciences (1/8, 12.5%), Engineering (1/8, 12.5%), Life Sciences (1/8, 12.5%), Medicine (2/8, 25%), Clinical Neurosciences (2/8, 25%) and the Auditory Implant Service (1/8, 12.5%). The researcher respondents held a mix of positions which included academic (1/8, 12.5%), clinician (2/8, 25%), academic and clinician (3/8, 37.5%), undergraduate (1/8, 12.5%) and project manager (1/8, 12.5%).

**Fig. 5:**
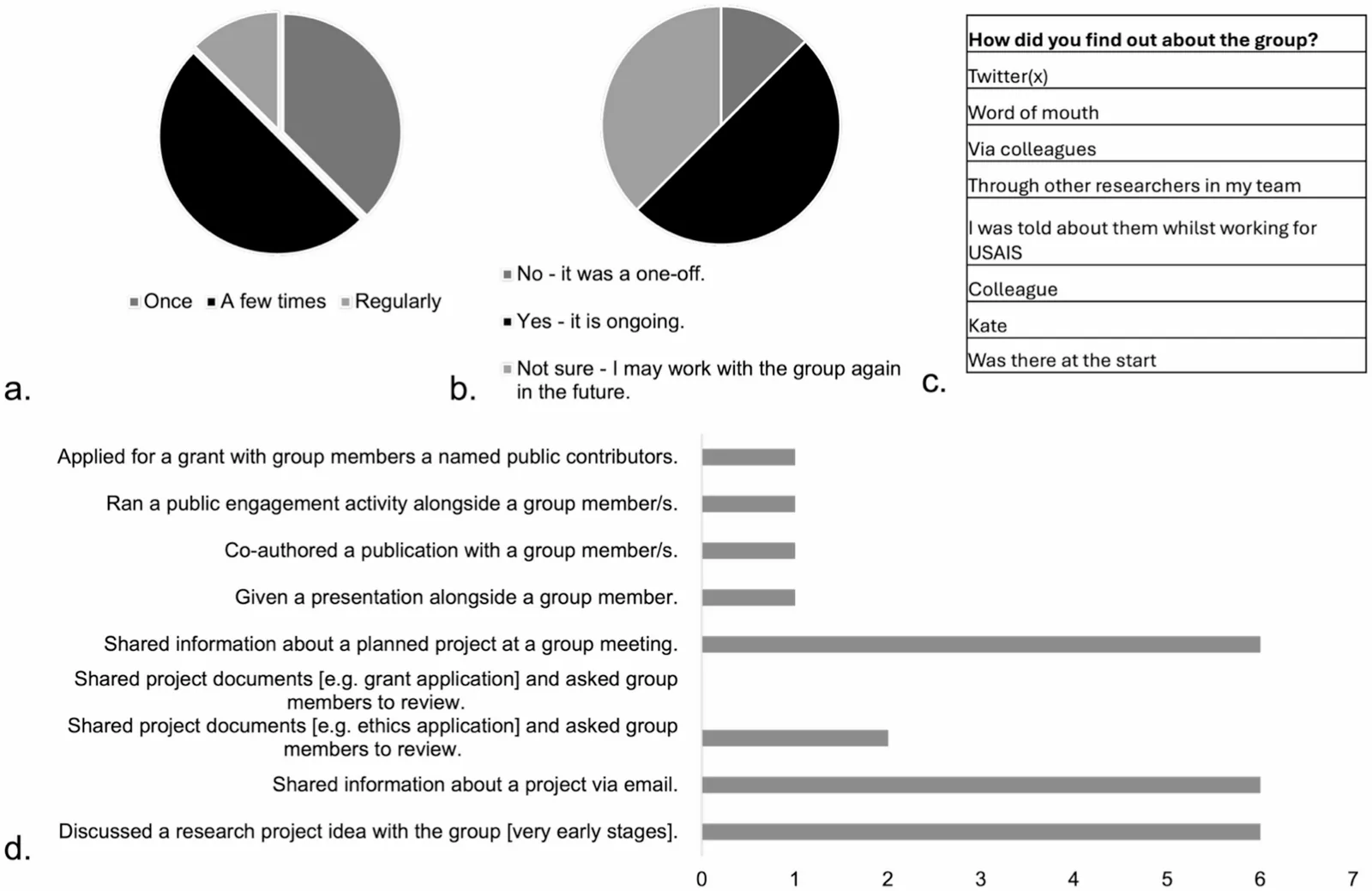
Summary of data regarding types of involvement from researchers with the group. The questions were: **a** How many times have you been involved with the group?. **b** Is your involvement with the group ongoing?

The majority of researchers (Fig. 5) have been involved with the group more than once (5/8, 62.5%) and their involvement is either ongoing (4/8, 50%) or may continue in the future (3/8, 37.5%). Most researcher respondents found out about the group through colleagues and other researchers (Fig. 5c). This highlights the importance and value of the All_Ears team in engaging with the research community to raise awareness of the group. Figure 5d demonstrates the variety of ways the researcher respondents have been involved with the group. The majority of respondents discussed early ideas for research projects and shared information about projects through emails and by attending in-person meetings.

### Rating scale questions

**Fig. 6:**
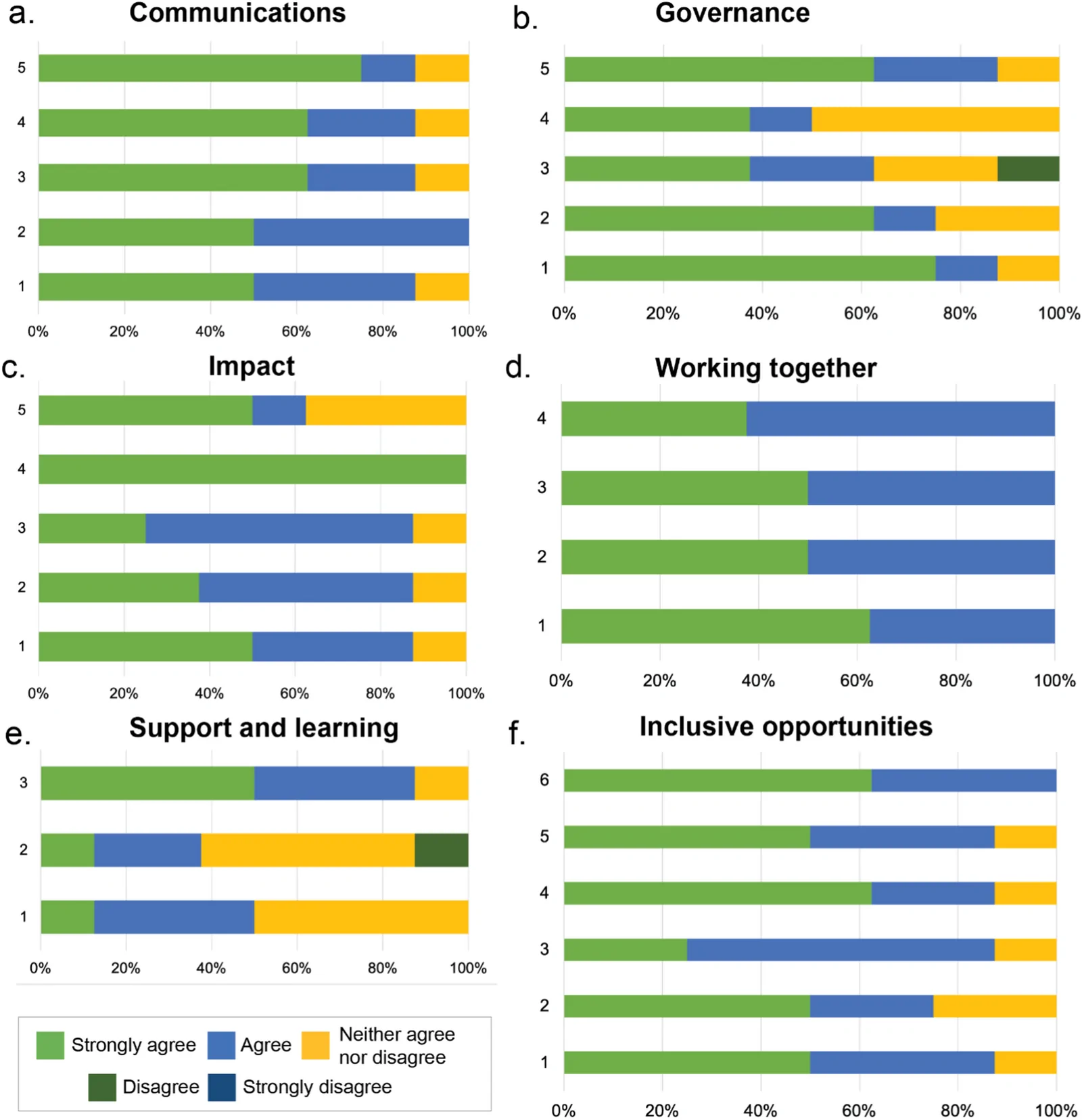
Stacked bar charts showing the rating scale question scores (%) from the researchers’ survey. Statements (y-axis) which aligned with each of the six UK Standards for Public Involvement were included. The numbers beside the bar summarise each statement within the standard and match the numbers listed next to the statements written in full in Table 6 and additional file 5c and 5d

Researchers strongly agreed there was value to working with All_Ears and that this had been beneficial for their research. Figure 6 summarises the scores (%) from the rating scale questions for the statements (y-axis) for each of the UK Standards for Public Involvement. Table 6 includes the statements which align with each of the UK Standards for Public Involvement. The number next to each statement in the table corresponds to the numbers in Fig. 6. See additional file 5c and 5d to visualise the scores alongside each of the statements. There were positive responses to the majority of the statements (76%, 22/29). ‘Working together’, was identified as a strength, all researchers either strongly agreed or agreed with this statement. This shows that the researchers acknowledge there is a collaborative approach between group members and researchers, that decisions are shared between group members, co-leads and researchers working with the group. ‘Communications’, ‘Impact’ and ’Inclusive opportunities’ received positive responses. For 22 out of 29 statements, at least one researcher selected at midpoint (neither agree nor disagree).

**Table 6:** Statements that were used for the rating scale questions in the researchers’ survey

UK Standards for Public Involvement						
Communications	There are/were clear and informative communications about involvement opportunities, upcoming meetings, written work, and activities (1).	The communications methods are inclusive (2).	The communications methods between myself as a researcher and the group members are/were flexible (3).	There are/were opportunities to share information, feedback and involvement opportunities with group members (4).	Information related to my research/project is/was shared with the group in a timely manner (5).	My research activities, learning and achievements (positive and negative) are/were shared amongst the group and more widely (6).
Governance	ALL_EARS@UoS group member’s voices are heard, valued, and respected in decision making (1).	Public involvement plans are in place and these plans are regularly monitored, reviewed, and reported on (2).	Throughout the University of Southampton, there is visible and accountable responsibility for public involvement (3).	Realistic resources such as money, staff, time are allocated to support the running of ALL_EARS@UoS (4).	The privacy of personal information is protected by collecting and using it in a suitable way (5).	
Impact	Group members are involved in deciding how the impact of their involvement should be assessed (1).	Time and activities such as meetings are allocated to reflect and evaluate the patient and public involvement (2).	I provide information to help the ALL_EARS@UoS team assess the impact of the group member’s involvement in my research such as who has been involved and how they were involved (3).	Working with ALL_EARS@UoS has been beneficial for my research (4).	My work with ALL_EARS@UoS has supported grant applications (5).	
Working together	The practical arrangements, preferences, and requirements for working together have been discussed, agreed between ALL_EARS@UoS co-lead, myself and group members (1).	It is clear that activities and plans have been developed as a group (2).	There is a shared understanding of roles, responsibilities, and expectations for patient and public involvement between group members and researchers (3).	Group member’s ideas, contributions and influence are recognised by researchers who have worked with the group and shared with the PPIE co-leads to be shared back to group members (4).		
Support and learning	A range of support and training is offered to group members and researchers to address identified needs (1).	Resources have been designated by ALL_EARS@UoS, through external funding, to support learning and development opportunities for both researchers, contributors, and staff (2).	There is a culture of learning by doing, building upon and sharing best practice between the whole group/team (3).			
Inclusive opportunities	PPIE group members are involved in research projects from the earliest stages (1).	Barriers to involvement such as payment or accessibility have been identified and addressed (2).	Information about activities is shared widely in ways that are accessible and appeal to different communities (3).	There are fair and transparent processes for ALL_EARS@UoS members to get involved with hearing research (4).	There is choice in the opportunities offered to PPIE group members (5).	The processes we have for members of the public to get involved with ALL_EARS@UoS results in an inclusive and diverse group (6).

Areas identified as needing improvement were the need for increased visibility of public involvement at the University and the need for better resources and support and learning for the group. One respondent disagreed with the statement ‘Throughout the University of Southampton, there is visible and accountable responsibility for public involvement’. Another respondent disagreed with the statement ‘Resources have been designated by All_Ears@UoS, through external funding, to support learning and development opportunities for both researchers, contributors, and staff’. Both group members and researchers identified Support and learning as areas for improvement. The UK Standard for Support and learning states that we should ’offer and promote support and learning opportunities that build confidence and skills for public involvement in research’. We need to invest time and designate resources through funding to develop and provide training opportunities for group members and researchers to develop their skillset to enable more involvement in research.

### Thematic analysis of open-text questions

Key themes generated from the reflexive thematic analysis of the responses from the open-text questions in the researchers’ survey were; benefits of working with the group for researchers [1]; positive impact on research and society [2]; value of group members [3]; and organisation and running of the group [4]. Each theme is comprised of subthemes (see Table 7). Additional file 6 outlines the full quotes from the free-text responses which align with the themes and subthemes. Participant identifiers for researchers are indicated in square brackets e.g. [R1].

**Table 7:** Themes and subthemes developed from the researchers’ survey responses

Theme	Subtheme	The UK Standard for Public Involvement that most aligns with each subtheme
1. Benefits of working with the group for researchers	1.1. Increased knowledge, awareness and skills 1.2 Value of working with the group members 1.3 Increased positive feeling from working with the group	Impact
		Impact
		Impact
2. Positive impact on research and society	2.1 Public involvement is an invaluable part of research 2.2 Improved study design, increased relevance and real-world applicability of research 2.3 Societal impact	Impact
		Impact
		Impact
3. Value of group members	3.1 Valuable contributions from group members 3.2 Positive attributes of group members	Impact
		Support and learning, Inclusive opportunities
4. Organisation and running of the group	4.1 Areas that are working well 4.2 Areas for improvement	Inclusive opportunities, Working together
		Working together

### Theme 1: Benefits of working with the group for researchers

The researchers described a range of benefits of working with the group such as gaining new, and improving existing, skills such as effective communication and acquiring new knowledge of PPIE.

#### Subtheme 1.1: Increased knowledge, awareness and skills

The researchers highlighted the importance and value of learning from those with lived experience to gain deeper insights into hearing loss. One researcher commented that it has helped them ‘develop their (my) knowledge and insight into PPIE and why it is important [R4]’ and another commented that this insight can ‘increase appropriate provision of clinical support [R3]’.

#### Subtheme 1.2: Value of working with the group members

The value of working with the group members included working with a diverse PPIE group and an ‘interested and valuable population [R5]’.

#### Subtheme 1.3: Increased positive feeling from working with the group

Benefits for the researchers of working with the group included that it ‘boosted (their) motivation and sense of purpose in their (my) research [R1]’ and that working with the group ‘increased (their) confidence in engaging the public meaningfully [R1]’.

### Theme 2: Positive impact on research and society

The researchers reflected on their perceived value and positive impact of the group on research and wider society.

#### Subtheme 2.1: Public involvement is an invaluable part of research

The perceived value of public involvement as part of the research process was highlighted and researchers described public involvement as a ‘valuable part of the research process [R1]’ or ‘an invaluable part of research [R4]’ and one researcher felt ‘the group has had invaluable contributions to their (my) research including allowing them (me) to gain more of an insight into hearing loss and helping to design studies that is most likely to benefit the people who it most affects [R4]’.

#### Subtheme 2.2: Improved study design, increased relevance and real-world applicability of research

Multiple benefits of incorporating public involvement at all stages of the research cycle were described by researchers such as improved study design, increased relevance and real-world applicability of research. One researcher stated that All_Ears ‘contributes to research at all stages [R4]’ and another researcher indicated that they worked with the group during the early stages of the research process. Four researchers described an impact of working with the group was increased relevance and therefore ‘Better-designed studies with real-world applicability [R1]’. One researcher explained that public involvement ‘means that research becomes relevant and focused on the users of cochlear implants and hearing technology [R2]’ and that ‘this has impact on national research and also society [R2]’.

#### Subtheme 2.3: Societal impact

Societal impact was highlighted which included ‘opportunity(ies) to influence real-world change [R1]’ and to ‘impact (on) national research and also society [R2]’. A key driver of All_Ears is to raise awareness of hearing and cochlear implants in the community. An increase in reach into the community was highlighted by one researcher.

### Theme 3: Value of group members

The researchers valued the input and insights from the group members through them sharing their lived experience and providing feedback.

#### Subtheme 3.1: Valuable contributions from group members

The researchers described the value of ‘constructive feedback [R1]’ from group members and described how ‘It is invaluable to have insights and guidance of CI users and those with hearing impairment [R2]’.

#### Subtheme 3.2: Positive attributes of group members

The researchers highlighted that group members were ‘very engaged [R7]’, ‘exceptional [R2] and a ‘brilliantly diverse, skilled group of individuals [R8]’.

### Theme 4: Organisation and running of the group

The diversity within the group, ‘inclusive atmosphere where all voices are heard and lived experiences are valued [R1]’, and good collaboration between group members and researchers which is facilitated by ‘clear communication and coordination from the group facilitators [R1]’ showed positivity from researchers about the organisation of the group.

#### Subtheme 4.1: Areas that are working well

‘Good communication between researchers and (the) group [R5]’ and ‘continuation of contact [R3]’ were reported as strengths which supported the running of the group. One researcher commented on the importance of having an approachable co-lead – ‘Probably the main success of this group is the very engaged and approachable and committed lead – Kate [R7]’.

#### Subtheme 4.2: Areas for improvement

The scheduling of meetings was identified as an area for improvement. One researcher suggested that ‘more frequent meetings [R2]’ would be helpful and another suggested that fixed scheduling of meetings may improve how researchers work with the group. One researcher commented that ‘time and funding are always the issues [R8]’, that ‘this is such an amazing group to work with but sometimes it would be great to have longer with them [R8]’.

One researcher commented that ‘more structured follow-up to show how public feedback has influenced research [R1]’ would improve how researchers work with the group. Completing the feedback cycle in public involvement is important as it ensures group members understand the impact of their involvement and know their feedback/input has been acknowledged and taken on board [25, 26]. When we start working with a new researcher, we highlight expectations around feedback and share our framework (see additional file 4 in [11]). When group members provide feedback on project documents or ideas, the researcher must provide feedback to the group members within two weeks. If the feedback cycle is not complete, there is a risk that group members could feel their involvement was tokenistic and not genuine.

## Discussion

This evaluation of the All_Ears PPIE group, using the UK Standards for Public Involvement, has identified the strengths and areas for improvement from the perspective of group members and researchers three years into the life of the group. Group members identified strengths in the effective communication during and between meetings, the friendly, supportive environment and shared passion to raise awareness and make a difference and the inclusivity within the group. Areas for improvement were: the opportunities to improve one’s skill to enable more involvement; the reimbursement process and the flexibility of meeting times and format. Researchers reported the perceived value and impact of working with the group on them, their research and on society and highlighted the diversity and skill of the group members as strengths. Increased visibility of public involvement at the University, investment of more resources to support training and learning and changes to the scheduling of meetings were suggested areas for improvement. Learnings from this evaluation may be useful and applicable for researchers who are setting up or running similar PPIE programmes.

When evaluating PPIE, it is important to disentangle the meaning of value and impact. The value of PPIE are the benefits of researchers and the public working in partnership in research. The impact of PPIE refers to the difference or changes that happen because of this partnership. These changes can be to the research, to the people involved, to the PPIE processes, to healthcare and to society. A key benefit of PPI for both group members and researchers is being valued. This was highlighted in our findings and in a recent scoping review of the value of PPIE on health research [24]. Researchers reported that sharing their ideas with group members increased their sense of purpose and made them feel like their research was valued. For the group members, feeling valued and feeling like their experiences and expertise were valued was important to them particularly for those who felt like their deafness often left them feeling undervalued. All researcher respondents felt strongly that working with All_Ears was beneficial for their research. By group members sharing their lived experiences, the researchers gained valuable insight and guidance which informed their research. Without a secure and trusting relationship, neither group members nor researchers are likely to feel valued for their role.

Increased confidence, knowledge and skills are impacts of public involvement [12, 24]. Group members and researchers described their perceived impact of raising awareness of deafness and the challenges that deaf and hard of hearing people face on the deaf community and on society. The impacts of PPIE on the research process and outputs are widely reported [4, 27, 28]. Despite this, some All_Ears group members were less clear about the impact of the work on research. This may be because members do not have enough ongoing feedback from researchers. Sharing the findings from the researchers’ survey with group members may support this. In addition, the timeline of taking a piece of research from idea to change in practice is typically many years. Better translation of the impact group members make on research is required.

The variety in PPIE activities and lack of reporting of impact is an issue in public involvement [13]. In a narrative review of reviews, the impacts that were reported were classified into four broad types (1) People (PPIE contributors, researchers, healthcare staff and policy makers), (2) Different phases of the research process, (3) Services and systems and (4) On the PPIE processes [13]. Like our findings, commonly documented impacts of PPIE related to the impacts on group members such as learning new skills and feeling empowered, while researchers reported improved understanding of, and collaboration with, the public. Researchers also described the impact of working with the public on the earlier phases of the research process [13]. Related to services and systems, one study described the positive impact at a community level through increased health literacy and better access to care and social support was described, which resonated with our work [29].

Several studies have described the emotional effort and difficulty that group members experience from sharing their personal [30–32] and sometimes painful experiences [24, 33] as running in parallel with the benefits. A negative impact on group members’ physical and mental health as a result of their involvement and the demand of the roles has been described. This was not highlighted by group members in our survey however a couple of members have had to leave the group due to increased caring and other responsibilities highlighting that a level of commitment is required. Many public contributors carry out their roles alongside employment, caring responsibilities and other community roles or responsibilities as well as living with health conditions. The possible physical, emotional and intellectual burden of public involvement for group members should be considered [34] and should be part of the expectation setting with researchers as they work to incorporate PPIE into their research.

The burdens of emotional labour for researchers such as feeling a responsibility to take care of, and support, the group members whilst dealing with their own emotions related to involvement has been described [33, 35]. Some researchers have described the challenge of having to be polite when they felt they wanted to be otherwise and suppressing their own opinions in front of public contributors [36]. This issue was not highlighted in our findings. If relationships are built where both parties feel they are on a level playing field, and a space is created for open and honest discussion, researchers should not feel like they have to watch what they say. Sometimes hard, confronting conversations are needed between researchers and group members for progress to be made. We have found that emotive topics can lead to heated discussions but with trust and careful facilitation, both researchers and group members can feel heard, listened to and satisfied with the outcome of the discussion [2]. Emotional labour for those involved in public involvement is inevitable as it involves people being vulnerable and sharing their personal experiences. It is important that this is acknowledged and that people are supported if needed.

The need for additional training is a recurring issue in public involvement [12, 37, 38]. The public and researchers have described feeling insufficiently equipped for collaboration and that they would like further training to support their involvement [12, 39]. This aligns with our findings. Different approaches to public involvement training, from group and peer-to-peer support [40] to more structured training for public contributors [41] have been described. One group trialled a group and peer-to-peer support programme where the goal was to learn from each other whilst collaborating on research projects [40]. Training tailored to public partners should teach about the research cycle and timelines to change in practice alongside highlighting what public involvement truly means and how public partners can meaningfully contribute to each stage of the research cycle. For researchers, embedding formal and experiential PPI training early on is beneficial for developing researchers’ skill set and has been shown to shift the focus of doctoral research projects resulting in more translatable research [42, 43]. Guidance materials have been developed which aim to support researchers who are engaging with the public in PPI [44, 45]. We will discuss training with group members and researchers to better understand what they feel could better support them and aim to implement this.

Many studies have only evaluated the value, impact and benefits from the public contributors’ perspective [12] and the experience of PPIE for researchers has been overlooked [36]. For non-tokenistic public involvement to be embedded within research and widespread, it is important that researchers appreciate the value for them and their research. Therefore, better evidence from evaluating researchers to understand the value and challenges for them is necessary [36, 46]. Our findings highlight the benefits to researchers of working with the group, the perceived impact on research and society, and add to the evidence base. Evaluating both researchers and group members allows us to understand how collaborative and meaningful the partnership is [47].

A priority for us is that All_Ears is diverse, inclusive and accepting. Diversity in the way group members think is important too, as we want group members to feel they can share their experiences and challenge views freely. By increasing the accessibility of our PPIE activities and reducing barriers to involvement, we aimed to support involvement from a wide range of people with diverse backgrounds and experiences [48]. Findings from the initial evaluation highlighted a need to improve the diversity of the group and to increase how inclusive and accessible the meetings and communications were. A consistent effort has been invested in both areas over the past two years and the findings from this evaluation reflect the progress we have made. Group members acknowledged the diversity of the group and highlighted the efforts the team have gone to, to engage with diverse people in terms of age, ethnicity, gender and type of hearing loss. Several researchers commented that the diversity of the group was a strength. Despite this, there are still communities that are currently not represented in the group such as members of the Deaf community who communicate predominantly using sign language. We commit to investing time and efforts to build trust with this community.

Another group currently not well represented in All_Ears is young people. Despite the examples of excellent initiatives where researchers have co-developed their activities and modes of working with young people [49–51], young people are a highly underrepresented group within public involvement [52]. Additional barriers that exist when working with young people such as lack of transport, limited availability due to education and digital exclusion [53, 54] often reduce or limit involvement of young people in research. To integrate the voice of young people in All_Ears, we established All_Ears@UoS Young People’s Advisory Group (YPAG) in March 2025, and we held our first meeting later that year. Our aim was to build a group for young people aged 14–16 years old with lived experience of deafness. We run (mostly) online meetings to increase the reach of the group and remove potential barriers to involvement such as location and travel. Recruitment has been a challenge, and we are still actively recruiting members (https://generic.wordpress.soton.ac.uk/all-ears/2025/03/07/join-our-new-young-peoples-advisory-group-ypag/). More targeted engagement with parents of young people with cochlear implants may help with recruitment. This group is in its early stages, and we look forward to building and growing a partnership with members of the YPAG.

It is important that PPIE group leads do not get complacent regarding diversity and inclusivity. We have found that carrying out community engagement alongside our public involvement activities has multiple benefits. Cochlear implants can be a life changing intervention for people with severe to profound hearing loss. Unfortunately, only a small percentage of those who could benefit from an implant actually has one [55, 56]. While this may reflect a personal choice not to have a device, people from underserved communities including those living in more deprived areas and those from ethnic minorities are less likely to be referred for cochlear implantation [12]. Through our consistent efforts to engage with local communities, we have not only increased the diversity of our group, but our group members are equally motivated to raise awareness and share knowledge within their communities – creating a ripple effect [23]. Combining both approaches has been effective in raising awareness in the local community and encouraging people from diverse backgrounds to get involved with the group. We encourage researchers and group leads who are developing similar programmes to carry out community engagement whilst establishing and running their public involvement activities.

Key strengths of this work are the co-developed evaluation surveys, and that we have evaluated both sides of the PPI partnership by gathering the perspectives of researchers and group members. The UK Standards for Public Involvement were used as a framework for the survey [14, 15, 17, 57]. To evaluate changes in the attitudes of group member respondents between the first and second evaluation, we replicated the format of our initial evaluation and compared the data from the statements that were in both evaluations. Both quantitative data and qualitative data were gathered. Targeted emails were sent out to each researcher who has worked with All_Ears inviting them to complete the questionnaire. We achieved a high response rate for the researchers’ survey (73%, 8/11). The response rate for the group members’ survey was 40% (18/44). Typically, low response rates in researchers’ evaluations have been reported [12] and often public contributors show higher response rates.

A further strength of this PPIE programme is the close and trusting relationships that have been developed over the years between group members and the group co-leads. The trust that has been built has formed the foundation for open and honest conversations and supported partnership working. These relationships likely influenced the research process. The group co-lead (KH) carried out the reflexive, thematic analysis. Her position within the group and the interpersonal relationships with group members likely influenced how the data were interpreted. This would have provided valuable contextual understanding of the group, its members, how it is run and how researchers engage with the group. It may have also influenced assumptions about the meaning of the respondents’ comments and influenced the importance of particular themes. As the group members knew the co-lead well and knew she would analyse the data, there could have been social desirability and relationship-related bias within their responses whereby group members may have been reluctant to be critical. These factors have been important to consider when interpreting the findings.

There were several limitations of this work. One was that a high proportion of respondents selected neither agree nor disagree for the rating scale questions, making aspects of the data interpretation difficult. Survey fatigue, feeling less informed or connected to topics, and being newer to the group may have affected the responses [58]. Equally, despite the survey being co-developed it may be that the questions lacked nuance or how easily they could be interpreted. To reduce the likelihood of this issue in future evaluations, it will be important to ask group members a question to determine their level of involvement i.e. how many online and in-person meetings they have attended and whether they regularly read meeting notes. Additionally, as this is an evaluation of one established PPIE programme in one institutional and geographical context, this may limit the transferability of the findings from this evaluation for other researchers and PPIE professionals. Finally, as 18/44 group members responded and 8/11 of the researchers responded to the survey, there was a possibility of non-response bias whereby those who chose to complete the survey differed from those who did not respond. Members who were more engaged with All_Ears, or who had particularly positive or negative experiences, may have been more likely to respond which could have influenced the overall findings.

## Conclusion

This evaluation has highlighted the perceived value and impact of the group from both the group members’ and researchers’ perspective. Increased knowledge and confidence and contributing to research were some of the benefits described by group members. Researchers felt there was value to working with All_Ears and that it was beneficial for their skillset, motivation and for their research. Researchers described that working with All_Ears improved the study design and increased the relevance and real-world applicability of their research. Overall, respondents’ experiences were positive across several of the UK Standards for Public Involvement while Support and learning and Governance were identified as areas requiring further development. Both group members and researchers recognised the great value of the diverse and inclusive group. Despite this, work to expand community representation is needed. A strength of this evaluation was that we gathered insight from researchers who have worked with the group and have a better understanding of what is useful for their research and what changes we can make to better facilitate their engagement with the group. Findings from this evaluation will be discussed with the group to develop an action plan for the future. As the reach and membership of All_Ears continues to grow and evolve over time, we look forward to experiencing the value and impact of the group on the members and researchers, on research and on society as a whole.

## Supplementary Information

Below is the link to the electronic supplementary material.

Supplementary Material 1

Supplementary Material 2

Supplementary Material 3

Supplementary Material 4

Supplementary Material 5

Supplementary Material 6

Supplementary Material 7

## Data Availability

The datasets generated and analysed during the current study are available in the University of Southampton PURE repository, [https://doi.org/10.5258/SOTON/D3955]. Note - The link will remain inactive until the paper has been accepted for publication.

## Declarations

### Authors’ information

KH is a post-doctoral research fellow in Faculty of Medicine and PPIE group co-lead for All_Ears. SS, MH, RB are members of All_Ears and were involved in the design stages of the evaluation. TN is a Professor in Neuroimmunology and Director of the University of Southampton Doctoral College.

## Abbreviations

BSL: British Sign Language
BMJ: British Medical Journal
CIICA: Cochlear implant international community of action
GRIPP2: Guidance for Reporting Involvement of Patients and the Public
IMD: Index of Multiple of Deprivation
SO: Southampton
LSOAs: Lower Layer Super Output Areas
PPI: Patient and public involvement
PPIE: Patient and public involvement and engagement
UK: United Kingdom
USAIS: University of Southampton Auditory Implant Service
YPAG: Young People’s Advisory Group
