# Supplementary material for: “It gives me confidence and value”: evaluating All_Ears@UoS patient and public involvement and engagement (PPIE) group three years on
Source: Res Involv Engagem. 2026 Oct 8;12:165. doi: 10.1186/s40900-026-00986-2 (PMC13647862; doi:10.1186/s40900-026-00986-2)
Supplement: Supplementary file 1 [file 40900_2026_986_MOESM1_ESM.docx]

**Additional file 1a. Group members’ survey.**

**Patient and Public Involvement and Engagement Evaluation – ALL_EARS@UoS**

**Introduction:**

It is now **three** **years** since we first advertised for people with lived experience of hearing loss and cochlear implants to work with researchers to support hearing research. This grew to become ALL_EARS@UoS. We have held 15 meetings since then, had various engagements with the wider community and now have around 55 members in the group.

We carried out our first formal evaluation between March – May 2023 and the findings from this informed our action plan. As the group reaches its third birthday, we want to reflect on and evaluate what is working well and what we need to improve. We have used the UK Standards for Public Involvement as a framework to design this questionnaire. The combination of short and longer answer questions should enable us to collect the breadth of information needed. To evaluate the diversity of the group, we will collect demographic information including age, gender, ethnicity and employment status and sector and first half of your postcode. We need to collect this information to show the progress we are making in involving the full range of people involved through both University of Southampton Auditory Implant Service (USAIS) and in the region the clinic covers.

The data will be used to evaluate what has happened so far and to produce an action plan for the coming 18 months and beyond. The questionnaire is anonymous so that we can share our findings with other support groups, clinical centres, and researchers. This may include a peer reviewed research paper which will describe the development of ALL_EARS@UoS, our progress and our future aims and goals.

This work will ensure that ALL_EARS@UoS is relevant for its members, for researchers working with the group and will be used to support continued funding for this activity.

**Section 1**

**Combined Participant Information Sheet and Consent Form for Anonymous Online Surveys**

Before completing this questionnaire, please click here to read the combined participant information sheet and consent form. The document will open in a new tab. Once you have read the document, click back on the Microsoft Forms tab to continue. https://acrobat.adobe.com/id/urn:aaid:sc:EU:6132de42-58b5-4009-91c8-65908f67e2ff

Before completing this questionnaire, please click here to read the combined participant information sheet and consent form. The document will open in a new tab. Once you have read the document, click back on the Microsoft Forms tab to continue. https://acrobat.adobe.com/id/urn:aaid:sc:EU:c49e078c-654a-446c-811b-03015b614484

I confirm that have read and understood information on this form, I am aged 18 or over and I agree to take part in this survey.

- Yes
- No

I confirm that I am happy to participate in this research.

- Yes
- No

I confirm that I happy for the data collected to be used for evaluation of ALL_EARS@UoS PPIE group.

- Yes
- No

I confirm that I am happy for the fully anonymised data collected to be shared through publications, social media, the university and with other clinical centres.

- Yes
- No

**Section 2**

**Statements related to UK Standards of Public Involvement**

The National Institute for Health and Care Research (NIHR) is the British government’s major funder of clinical, public health, social care, and translational research. It is the nation’s largest funder of health and care research, providing the people, facilities, and technology for research to thrive.

**The NIHR have an established set of Standards for Public Involvement which cover six areas. The standards are below.**

**Communications:** use plain language for well-timed and relevant communications, as part of involvement plans and activities.

**Governance:** involve the public in research management, regulation, leadership, and decision making

**Impact:** seek improvement by identifying and sharing the difference that public involvement makes to research

**Working together:** work together in a way that values all contributions and builds and sustains mutually respectful and productive relationships.

**Support and learning:** offer and promote support and learning opportunities that build confidence and skills for public involvement in research.

**Inclusive opportunities:** offer public involvement opportunities that are accessible and that reach people and groups according to research needs.

Using these standards, we have put together some questions to get an idea of how we are doing in each area of the standards.

***Please indicate how strongly you agree or disagree with the following statements.***

Section 3

**Communications**

UK Standard for Public Involvement - **Communications:** use plain language for well-timed and relevant communications, as part of involvement plans and activities.

*There are clear and informative communications about upcoming meetings, written work, and activities.*

Strongly agree

Agree

Neither agree nor disagree

Disagree

Strongly disagree

*The methods for communicating in meetings and via emails are inclusive for the whole group.*

Strongly agree

Agree

Neither agree nor disagree

Disagree

Strongly disagree

*The methods for communicating in meetings and via emails are flexible for the whole group i.e group members can communicate with ALL_EARS@UoS co-leads a number of ways such as via email, on video calls, my text, my letters in the post.*

Strongly agree

Agree

Neither agree nor disagree

Disagree

Strongly disagree

*There are regular opportunities to offer feedback about meetings, project ideas and activities.*

Strongly agree

Agree

Neither agree nor disagree

Disagree

Strongly disagree

*My feedback is gathered, acted on and shared back to the group.*

Strongly agree

Agree

Neither agree nor disagree

Disagree

Strongly disagree

*Our activities, learning and achievements (positive and negative) are shared amongst and the group and more widely.*

Strongly agree

Agree

Neither agree nor disagree

Disagree

Strongly disagree

If you would like to add comments to clarify your answers related to ‘Communication’, please write your comments here. (To keep the survey anonymous, please do not include any information from which you, or other people, could be identified).

***Section 4***

**Governance**

UK Standard for Public Involvement: Governance - **Governance:** involve the public in research management, regulation, leadership, and decision making.

*Group member’s voices are heard, valued, and respected in decision making.*

Strongly agree

Agree

Neither agree nor disagree

Disagree

Strongly disagree

*Public involvement plans are in place and these plans are regularly monitored, reviewed, and reported on.*

Strongly agree

Agree

Neither agree nor disagree

Disagree

Strongly disagree

*Throughout the organisation, there is visible and accountable responsibility for public involvement.*

Strongly agree

Agree

Neither agree nor disagree

Disagree

Strongly disagree

*Realistic resources such as money, staff, time are allocated for public involvement.*

Strongly agree

Agree

Neither agree nor disagree

Disagree

Strongly disagree

*The privacy of personal information is protected by collecting and using it in a suitable way.*

Strongly agree

Agree

Neither agree nor disagree

Disagree

Strongly disagree

If you would like to add comments to clarify your answers related to ‘Governance’, please write your comments here. (To keep the survey anonymous, please do not include any information from which you, or other people, could be identified).

……………………………………………………………………….……………………………………………………………………….

……………………………………………………………………….……………………………………………………………………….

***Section 5***

**Impact**

**UK Standard for Public Involvement - Impact:** seek improvement by identifying and sharing the difference that public involvement makes to research.

*Group members are involved in deciding how the impact of their involvement should be assessed (I.e., Group members are involved in the process of evaluating impact).*

Strongly agree

Agree

Neither agree nor disagree

Disagree

Strongly disagree

*Information is collected to help assess impact of the involvement such as who has been involved and how they were involved.*

Strongly agree

Agree

Neither agree nor disagree

Disagree

Strongly disagree

*Time and activities such as meetings are allocated to reflect and evaluate the patient and public involvement.*

Strongly agree

Agree

Neither agree nor disagree

Disagree

Strongly disagree

*My involvement has an impact on research.*

Strongly agree

Agree

Neither agree nor disagree

Disagree

Strongly disagree

If you would like to add comments to clarify your answers related to ‘Impact’, please write your comments here. (To keep the survey anonymous, please do not include any information from which you, or other people, could be identified).

**Section 6**

**Working together**

UK Standard for Public Involvement - **Working together:** work together in a way that values all contributions and builds and sustains mutually respectful and productive relationships.

*The aims and purpose of the group have been jointly agreed and defined by the group.*

Strongly agree

Agree

Neither agree nor disagree

Disagree

Strongly disagree

*I feel valued and able to share ideas freely without restriction.*

Strongly agree

Agree

Neither agree nor disagree

Disagree

Strongly disagree

*The practical arrangements, preferences, and requirements for working together have been discussed, addressed, and agreed as a group.*

Strongly agree

Agree

Neither agree nor disagree

Disagree

Strongly disagree

*In activities, the team are clear about objectives, how to achieve them and how to present the results.*

Strongly agree

Agree

Neither agree nor disagree

Disagree

Strongly disagree

*There is a shared understanding of roles, responsibilities, and expectations for patient and public involvement.*

Strongly agree

Agree

Neither agree nor disagree

Disagree

Strongly disagree

*Our individual ideas and contributions are recognised, addressed and shared with the group.*

Strongly agree

Agree

Neither agree nor disagree

Disagree

Strongly disagree

If you would like to add comments to clarify your answers related to ‘Working together’, please write your comments here. (To keep the survey anonymous, please do not include any information from which you, or other people, could be identified).

……………………………………………………………………….……………………………………………………………………….

……………………………………………………………………….……………………………………………………………………….

……………………………………………………………………….……………………………………………………………………….

***Section 7***

**Support and learning**

UK Standard for Public Involvement - **Support and learning:** offer and promote support and learning opportunities that build confidence and skills for public involvement in research.

*There are opportunities to improve one’s skill set to enable more involvement.*

Strongly agree

Agree

Neither agree nor disagree

Disagree

Strongly disagree

*Resources have been designated to support learning and development opportunities for both researchers, ALL_EARS members, and staff.*

Strongly agree

Agree

Neither agree nor disagree

Disagree

Strongly disagree

*There is a culture of learning by doing, building upon and sharing best practice between the whole group/team.*

Strongly agree

Agree

Neither agree nor disagree

Disagree

Strongly disagree

If you would like to add comments to clarify your answers related to ‘Support and learning’, please write your comments here. (To keep the survey anonymous, please do not include any information from which you, or other people, could be identified).

……………………………………………………………………….……………………………………………………………………….

……………………………………………………………………….……………………………………………………………………….

……………………………………………………………………….……………………………………………………………………….

***Section 8***

**Inclusive opportunities**

UK Standard for Public Involvement - **Inclusive opportunities:** offer public involvement opportunities that are accessible and that reach people and groups according to research needs.

*Patient and public group members are involved in the research from the earliest stages.*

Strongly agree

Agree

Neither agree nor disagree

Disagree

Strongly disagree

*Barriers to involvement such as payment or accessibility have been identified and addressed.*

Strongly agree

Agree

Neither agree nor disagree

Disagree

Strongly disagree

*Information about activities is shared widely in ways that are accessible and appeal to different communities.*

Strongly agree

Agree

Neither agree nor disagree

Disagree

Strongly disagree

*There is choice in the opportunities that are offered for group members to get involved with research.*

Strongly agree

Agree

Neither agree nor disagree

Disagree

Strongly disagree

*The processes we have for getting involved result in an inclusive and diverse group.*

Strongly agree

Agree

Neither agree nor disagree

Disagree

Strongly disagree

*The group is a true representation of the Deaf or hard of hearing community.*

Strongly agree

Agree

Neither agree nor disagree

Disagree

Strongly disagree

If you would like to add comments to clarify your answers related to ‘Inclusive opportunities’, please write your comments here. (To keep the survey anonymous, please do not include any information from which you, or other people, could be identified).

……………………………………………………………………….……………………………………………………………………….

……………………………………………………………………….……………………………………………………………………….

……………………………………………………………………….……………………………………………………………………….

**Now is your opportunity to provide some written feedback.**

For the following free-text responses – to ensure the survey remains anonymous, please do not include any information from which you, or other people, could be identified.

**What has worked well?**

*Answer:* ……………………………………………………………………….……………………………………………………………………….

……………………………………………………………………….……………………………………………………………………….

……………………………………………………………………….……………………………………………………………………….

**What areas would you like to see improvements in?**

*Answer:*

……………………………………………………………………….……………………………………………………………………….

……………………………………………………………………….……………………………………………………………………….

……………………………………………………………………….……………………………………………………………………….

**What do you see as the impact of being involved within this group?**

You can comment on the impact on you, impact on others with hearing loss, impact on the research community and impact on wider society.

*Answer:*

……………………………………………………………………….……………………………………………………………………….

……………………………………………………………………….……………………………………………………………………….

……………………………………………………………………….……………………………………………………………………….

**Describe any benefits of being part of ALL_EARS.**

*Answer:*

……………………………………………………………………….……………………………………………………………………….

……………………………………………………………………….……………………………………………………………………….

……………………………………………………………………….……………………………………………………………………….

**Now is your opportunity to reflect on your engagement with the group.**

On a scale of 1 to five, how much do you feel you have contributed to the group?

*1 is low contribution, 5 is greatest contribution.*

1	2	3	4	5

Please add some examples of your engagement, if relevant.

**Do you think you are likely to be involved in a year’s time?**

……………………………………………………………………….……………………………………………………………………….

If there is anything you would like to add regarding future actions for ALL_EARS@UoS, please include below:

……………………………………………………………………….……………………………………………………………………….

……………………………………………………………………….……………………………………………………………………….

……………………………………………………………………….……………………………………………………………………….

**Section 3**

**Demographic data**

This data is so we can evaluate the reach of ALL_EARS@UoS relative to the population of people at USAIS and in the geographical region covered by the clinic.

**Which category below includes your age?**

17 or younger

18-29

30-49

50-69

70-79

80 - or older

Prefer not to say

**To which gender identity do you most identify?**

Woman

Man

Non-binary

Not listed

Prefer not to say

If your gender identity is not listed, please write it below.

**Ethnicity**

**What is your ethnic group? Choose one option that best describes your ethnic group or background.**

**Asian or Asian British**

- Indian
- Pakistani
- Bangladeshi
- Chinese
- Any other Asian background

**Black, Black British, Caribbean or African**

- Caribbean
- African
- Any other Black, Black British, or Caribbean background

**Mixed or multiple ethnic groups**

- White and Black Caribbean
- White and Black African
- White and Asian
- Any other Mixed or multiple ethnic background

**White**

- English, Welsh, Scottish, Northern Irish or British
- Irish
- Gypsy or Irish Traveller
- Roma
- Any other White background

**Other ethnic group**

- Arab
- Any other ethnic group

Prefer not to say

**What is the first half of your postcode?**

**Employment**

**Which of the following categories best describes your employment status?** Please tick all that apply.

Employed, full time

Employed, part-time

Full time carer

Not employed, looking for work

Not employed, NOT looking for work

Retired – with several community commitments e.g. All_Ears, member of school or other community board

Retired – community commitment All_Ears

Disabled, not able to work

**What is or was (if you are now retired) your main job sector? E.g. Marketing, Hospitality, Healthcare.**

…………………………………..

**What is your main language?**

- English
- Other, please describe:

If you selected other for Q8, please describe below.

**What is your lived experience of hearing loss/deafness? Please select the option/(s) which most suit your experience. Or write your own description below.**

I am deaf.

I am partially deaf.

I have mild hearing loss or deafness.

I have moderate hearing loss or deafness.

I have severe hearing loss or deafness.

I have profound hearing loss or deafness.

A close family member or friend is D/deaf/has hearing loss/ is hard of hearing.

I live with someone who is deaf/has hearing loss.

None of the above

Or please write your own description here:

**If you are deaf or hard of hearing, do you use any interventions or assistive listening devices?**

Please select all of the statements which apply to you.

I wear one hearing aid.

I wear two hearing aids.

I have one cochlear implant.

I have two cochlear implants.

I connect to loop systems when they are available using my hearing aid and/or cochlear implant.

I use a Bluetooth microphone linked with my hearing aid or cochlear implant.

I use Speech-to-text on my device.

I use an assistive listening device (personal listener).

*I use a remote control to change the settings and/or* *connect to loop systems TV etc.*

I connect my cochlear implant to an app on my phone to manage settings.

I use Text Relay for the phone.

I use an interpreter.

Please describe any other approaches you may use.

________________

**How were you recruited into the group?**

Through University of Southampton Auditory Implant Service

Through community groups

Through seeing adverts online or on social media

Through finding out about the group through word of mouth

Researchers’ survey

**ALL_EARS@UoS Evaluation Survey for Researchers**

**Section 1**

**Patient and Public Involvement and Engagement Evaluation – ALL_EARS@UoS**

**Introduction:**

It is now **three** **years** since we first advertised for people with lived experience of hearing loss and cochlear implants to work with researchers to support hearing research. This grew to become ALL_EARS@UoS. We have held 15 meetings since then, had various engagements with the wider community and now have around 55 members in the group.

We carried out our first formal evaluation between March – May 2023 and the findings from this informed our action plan. As the group reaches its third birthday, we want to reflect on and evaluate what is working well and what we need to improve. We have used the UK Standards for Public Involvement as a framework to design this questionnaire. The combination of short and longer answer questions should enable us to collect the breadth and depth of information needed.

You are being asked to complete this questionnaire because you are a researcher and have worked with ALL_EARS@UoS PPIE group since the group was first established in March 2022. We want to understand your perspective and experience as a researcher, of working with ALL_EARS@UoS group during the period of March 2022 to now. We will be asking ALL_EARS@UoS group members for their perspective using a different questionnaire that follows a similar format.

The data will be used to evaluate what has happened so far and to produce an action plan for the coming 18 months and beyond. The questionnaire is anonymous so that we can share our findings with other support groups, clinical centres, and researchers. This may include a peer reviewed research paper which will describe the development of ALL_EARS@UoS, our progress and our future aims and goals.

This work will ensure that ALL_EARS@UoS is relevant for its members, for researchers working with the group and will be used to support continued funding for this activity.

**Section 1**

**Combined Participant Information Sheet and Consent Form for Anonymous Online Surveys**

Before completing this questionnaire, please click here to read the combined participant information sheet and consent form. The document will open in a new tab. Once you have read the document, click back on the Microsoft Forms tab to continue. <https://acrobat.adobe.com/id/urn:aaid:sc:EU:c49e078c-654a-446c-811b-03015b614484>

I confirm that have read and understood information on this form, I am aged 18 or over and I agree to take part in this survey.

- Yes
- No

I confirm that I am happy to participate in this research.

- Yes
- No

I confirm that I happy for the data collected to be used for evaluation of ALL_EARS@UoS PPIE group.

- Yes
- No

I confirm that I am happy for the fully anonymised data collected to be shared through publications, social media, the university and with other clinical centres.

- Yes
- No

**Section 2**

**Statements related to UK Standards of Public Involvement**

**The NIHR have an established set of Standards for Patient and Public Involvement which cover six areas. The standards are below.**

**Communications:** use plain language for well-timed and relevant communications, as part of involvement plans and activities.

**Governance:** involve the public in research management, regulation, leadership, and decision making.

**Impact:** seek improvement by identifying and sharing the difference that public involvement makes to research.

**Working together:** work together in a way that values all contributions and builds and sustains mutually respectful and productive relationships.

**Support and learning:** offer and promote support and learning opportunities that build confidence and skills for public involvement in research.

**Inclusive opportunities:** offer public involvement opportunities that are accessible and that reach people and groups according to research needs.

Using these standards, we have put together some statements to get an idea of how we are doing in each area of the standards.

***Please indicate how strongly you agree or disagree with the following statements.***

Section 3

**Communications**

UK Standard for Public Involvement - **Communications:** use plain language for well-timed and relevant communications, as part of involvement plans and activities.

*There are/were clear and informative communications about involvement opportunities, upcoming meetings, written work, and activities.*

Strongly agree

Agree

Neither agree nor disagree

Disagree

Strongly disagree

*The communications methods are inclusive.*

Strongly agree

Agree

Neither agree nor disagree

Disagree

Strongly disagree

*The communications methods between myself as a researcher and the group members are/were flexible (for example – via email, face-to-face discussion in group meetings, written/mailed documents).*

Strongly agree

Agree

Neither agree nor disagree

Disagree

Strongly disagree

*There are/were opportunities to share information, feedback and involvement opportunities with group members.*

Strongly agree

Agree

Neither agree nor disagree

Disagree

Strongly disagree

*Information related to my research/project is/was shared with the group in a timely manner.*

Strongly agree

Agree

Neither agree nor disagree

Disagree

Strongly disagree

*My research activities, learning and achievements (positive and negative) are/were shared amongst the group and more widely.*

Strongly agree

Agree

Neither agree nor disagree

Disagree

Strongly disagree

If you would like to add comments to clarify your answers related to ‘Communication’, please write your comments here. (To keep the survey anonymous, please do not include any information from which you, or other people, could be identified).

**Governance:**

*ALL_EARS@UoS group member’s voices are heard, valued, and respected in decision making.*

Strongly agree

Agree

Neither agree nor disagree

Disagree

Strongly disagree

*Public involvement plans are in place and these plans are regularly monitored, reviewed, and reported on.*

Strongly agree

Agree

Neither agree nor disagree

Disagree

Strongly disagree

*Throughout the University of Southampton, there is visible and accountable responsibility for public involvement.*

Strongly agree

Agree

Neither agree nor disagree

Disagree

Strongly disagree

*Realistic resources such as money, staff, time are allocated to support the running of ALL_EARS@UoS.*

Strongly agree

Agree

Neither agree nor disagree

Disagree

Strongly disagree

*The privacy of personal information is protected by collecting and using it in a suitable way.*

Strongly agree

Agree

Neither agree nor disagree

Disagree

Strongly disagree

If you would like to add comments to clarify your answers related to ‘Governance’, please write your comments here. (To keep the survey anonymous, please do not include any information from which you, or other people, could be identified).

**Impact:**

*Group members are involved in deciding how the impact of their involvement should be assessed (I.e., Group members are involved in deciding how to evaluate the progress and impact of the group).*

Strongly agree

Agree

Neither agree nor disagree

Disagree

Strongly disagree

*Time and activities such as meetings are allocated to reflect and evaluate the patient and public involvement.*

Strongly agree

Agree

Neither agree nor disagree

Disagree

Strongly disagree

*I provide information to help the ALL_EARS@UoS team assess the impact of the group member’s involvement in my research such as who has been involved and how they were involved.*

Strongly agree

Agree

Neither agree nor disagree

Disagree

Strongly disagree

Working with ALL_EARS@UoS has been beneficial for my research.

Strongly agree

Agree

Neither agree nor disagree

Disagree

Strongly disagree

My work with ALL_EARS@UoS has supported grant applications.

Strongly agree

Agree

Neither agree nor disagree

Disagree

Strongly disagree

If you would like to add comments to clarify your answers related to ‘Impact’, please write your comments here. (To keep the survey anonymous, please do not include any information from which you, or other people, could be identified).

**Working together:**

*The practical arrangements, preferences, and requirements for working together have been discussed, addressed, and agreed between ALL_EARS@UoS co-lead, myself and group members.*

Strongly agree

Agree

Neither agree nor disagree

Disagree

Strongly disagree

*It is clear that activities and plans have been developed as a group.*

Strongly agree

Agree

Neither agree nor disagree

Disagree

Strongly disagree

*There is a shared understanding of roles, responsibilities, and expectations for patient and public involvement between group members and researchers.*

Strongly agree

Agree

Neither agree nor disagree

Disagree

Strongly disagree

*Group member’s ideas, contributions and influence are recognised by researchers who have worked with the group and shared with the PPIE co-leads to be shared back to group members.*

Strongly agree

Agree

Neither agree nor disagree

Disagree

Strongly disagree

If you would like to add comments to clarify your answers related to ‘Working together’, please write your comments here.

**Support and learning:**

*A range of support and training is offered to group members and researchers to address identified needs.*

Strongly agree

Agree

Neither agree nor disagree

Disagree

Strongly disagree

*Resources have been designated by ALL_EARS@UoS, through external funding, to support learning and development opportunities for both researchers, contributors, and staff.*

Strongly agree

Agree

Neither agree nor disagree

Disagree

Strongly disagree

*There is a culture of learning by doing, building upon and sharing best practice between the whole group/team.*

Strongly agree

Agree

Neither agree nor disagree

Disagree

Strongly disagree

If you would like to add comments to clarify your answers related to ‘Support and learning’, please write your comments here. (To keep the survey anonymous, please do not include any information from which you, or other people, could be identified).

**Inclusive opportunities:**

*PPIE group members are involved in research projects from the earliest stages.*

Strongly agree

Agree

Neither agree nor disagree

Disagree

Strongly disagree

*Barriers to involvement such as payment or accessibility have been identified and addressed.*

Strongly agree

Agree

Neither agree nor disagree

Disagree

Strongly disagree

*Information about activities is shared widely in ways that are accessible and appeal to different communities.*

Strongly agree

Agree

Neither agree nor disagree

Disagree

Strongly disagree

*There are fair and transparent processes for ALL_EARS@UoS members to get involved with hearing research.*

Strongly agree

Agree

Neither agree nor disagree

Disagree

Strongly disagree

*There is choice in the opportunities offered to PPIE group members.*

Strongly agree

Agree

Neither agree nor disagree

Disagree

Strongly disagree

*The processes we have for members of the public to get involved with ALL_EARS@UoS results in an inclusive and diverse group.*

Strongly agree

Agree

Neither agree nor disagree

Disagree

Strongly disagree

If you would like to add comments to clarify your answers related to ‘Inclusive opportunities’, please write your comments here. (To keep the survey anonymous, please do not include any information from which you, or other people, could be identified).

**Now is your opportunity to provide some written feedback.** For the following free-text responses – to ensure the survey remains anonymous, please do not include any information from which you, or other people, could be identified.

**Q1. What has worked well?**

*Answer:*

**Q2. What areas would you like to see improvements in to improve how researchers work with ALL_EARS@UoS?**

*Answer:*

**Q3. What do you see as the impact of working with ALL_EARS@UoS?**

You can comment on the impact on you and your research, impact on others with hearing loss, impact on the research community and impact on wider society.

*Answer:*

**Q4. Describe any benefits of being part of ALL_EARS@UoS.**

*Answer:*

**Are you likely to work with ALL_EARS@UoS again in the future?**

- Yes
- No
- Not sure

If there is anything else you would like to add about your involvement with ALL_EARS@UoS, please include below.

**Section 3**

Demographic questions about the researchers:

**To which gender identity do you most identify?**

Woman

Man

Non-binary

Not listed ……………

Prefer not to say

If your gender identity is not listed, please write it below.

What institution are you based at?

What faculty within that institution do you belong to?

**What is your current position?**

Postgraduate researcher

Post doctoral researcher

Academic

Clinician

Other – please specify:

**What type of involvement have you had with ALL_EARS@UoS PPIE group?	[tick all that apply]**

Discussed a research project idea with the group [very early stages].

Shared information about a project via email.

Shared project documents [e.g. ethics application] and asked group members to review.

Shared project documents [e.g. grant application] and asked group members to review.

Shared information about a planned project at a group meeting.

Given a presentation alongside a group member.

Co-authored a publication with a group member/s.

Ran a public engagement activity alongside a group member/s.

Applied for a grant with group members a named public contributors.

**How did you find out about ALL_EARS@UoS?**

x

**How many times have you been involved with the group?**

Once

A few times (2-4)

Regularly

**Is your involvement with ALL_EARS@UoS group ongoing?**

No – it was a one-off.

Yes – it is ongoing.

Not sure – I may work with the group again the in the future.
