# Supplementary material for: “It gives me confidence and value”: evaluating All_Ears@UoS patient and public involvement and engagement (PPIE) group three years on
Source: Res Involv Engagem. 2026 Oct 8;12:165. doi: 10.1186/s40900-026-00986-2 (PMC13647862; doi:10.1186/s40900-026-00986-2)
Supplement: Supplementary file 2 [file 40900_2026_986_MOESM2_ESM.docx]

| **Section and topic** | **Item** | **Reported on page number** |
| --- | --- | --- |
| Section 1: Abstract of paper | | |
| 1a. Aim | Report the aim of the study | 2 |
| 1b. Methods | Describe the methods used by which patients and the public were involved | 2 |
| 1c. Results | Report the impacts and outcomes of PPI in the study | 2 |
| 1d: Conclusions | Summarise the main conclusions of the study | 2, 3 |
| 1e: Keywords | Include PPI, “patient and public involvement,” or alternative terms as keywords | 4 |
| **Section 2: Background to paper** | | |
| 2a: Definition | Report the definition of PPI used in the study and how it links to comparable studies. | 4 |
| 2b: Theoretical underpinnings | Report the theoretical rationale and any theoretical influences relating to PPI in the study. | 5 – 9 |
| 2c: Concepts and theory development | Report any conceptual or theoretical models, or influences, used in the study | UK Standards for Public Involvement - 9 |
| **Section 3: Aims of paper** | | |
| 3: Aim | Report the aim of the study | 10 |
| **Section 4: Methods of paper** | | |
| 4a: Design | Provide a clear description of methods by which patients and the public were involved. *(what did the PPI contributors do)* | Establishing the group - 6  Evaluation steering group: 10 |
| 4b: People involved | Provide a description of patients, carers, and the public involved with the PPI activity in the study. | 10 |
| 4c: Stages of involvement | Report on how PPI is used at different stages of the study | 10 (design), 12 (analysis), 13 (interpretation), 13 (dissemination) |
| 4d: Level or nature of involvement | Report the level or nature of PPI used at various stages of the study. | 10 |
| **Section 5: Capture or measurement of PPI impact** | | |
| 5a: Qualitative evidence of impact | If applicable, report the methods used to qualitatively explore the impact of PPI in the study | Rating scale questions, open-text questions – 11 |
| 5b: Quantitative evidence of impact | If applicable, report the methods used to quantitatively measure or assess the impact of PPI | Group attendance – 10 |
| 5c: Robustness of measure | If applicable, report the rigour of the method used to capture or measure the impact of PPI | 12, 13 |
| **Section 6: Economic assessment** | | |
| 6: Economic assessment | If applicable, report the method used for an economic assessment of PPI | N/a |
| **Section 7: Study results** | | |
| 7a: Outcomes of PPI | Report the results of PPI in the study, including both positive and negative outcomes*. (how did the PPI contribute/ to the study)* | 14 – 41 |
| 7b: Impacts of PPI | Report the positive and negative impacts that PPI has had on the research, the individuals involved (including patients and researchers), and wider impacts. | On research – 22, 23, 24, 25  35 - 41  On individuals involved –  25 – 30  37 - 41  Wider impact –  27, 28, 38, 39, 40 |
| 7c: Context of PPI | Report the influence of any contextual factors that enabled or hindered the process or impact of PPI. | Working with deaf and hard of hearing people – 5, 6 |
| 7d: Process of PPI | Report the influence of any process factors, that enabled or hindered the impact of PPI. | Running of the group –  6 – 8  30 – 32  40 - 51 |
| 7ei: Theory development | Report any conceptual or theoretical development in PPI that have emerged. | n/a |
| 7eii: Theory development | Report evaluation of theoretical models, if any | n/a |
| 7f: Measurement | If applicable, report all aspects of instrument development and testing (eg, validity, reliability, feasibility, acceptability, responsiveness, interpretability, appropriateness, precision) | n/a |
| 7g: Economic assessment | Report any information on the costs or benefit of PPI | n/a |
| **Section 8: Discussion and conclusions** | | |
| 8a: Outcomes | Comment on how PPI influenced the study overall. Describe positive and negative effects. | 42 - 49 |
| 8b: Impacts | Comment on the different impacts of PPI identified in this study and how they contribute to new knowledge. | 42, 43 |
| 8c: Definition | Comment on the definition of PPI used (reported in the Background section) and whether or not you would suggest any changes | n/a |
| 8d: Theoretical underpinnings | Comment on any way your study adds to the theoretical development of PPI. |  |
| 8e: Context | Comment on how context factors influenced PPI in the study | 42 - 47 |
| 8f: Process | Comment on how process factors influenced PPI in the study | 42 - 47 |
| 8g: Measurement and capture of PPI impact | If applicable, comment on how well PPI impact was evaluated or measured in the study. | 42 - 43 |
| 8h: Economic assessment: | If applicable, discuss any aspects of the economic cost or benefit of PPI, particularly any suggestions for future economic modelling. | n/a |
| 8i: Reflections/critical perspective | Comment critically on the study, reflecting on the things that went well and those that did not, so that others can learn from this study. | 47 – 49 |
