# Supplementary material for: “It gives me confidence and value”: evaluating All_Ears@UoS patient and public involvement and engagement (PPIE) group three years on
Source: Res Involv Engagem. 2026 Oct 8;12:165. doi: 10.1186/s40900-026-00986-2 (PMC13647862; doi:10.1186/s40900-026-00986-2)
Supplement: Supplementary file 3 [file 40900_2026_986_MOESM3_ESM.docx]

| **Number of meetings** | **Date of meeting** | **Total attendance** | **Breakdown of attendees** | | | |
| --- | --- | --- | --- | --- | --- | --- |
|  |  |  | **New members** | **Patients at USAIS** | **Recruited through adverts / word of mouth** | **Recruited from local community via community engagement** |
| 1 | May 2022 | 9 | 9 | 8 | 1 | 0 |
| 2 | June 2022 | 5 | 1 | 5 | 0 | 0 |
| 3 | September 2022 | 6 | 2 | 5 | 1 | 0 |
| 4 | November 2022 | 7 | 0 | 6 | 1 | 0 |
| 5 | January 2022 | 12 | 6 | 9 | 3 | 0 |
| 6 | May 2023 | 14 | 10 | 4 | 0 | 10 |
| 7 | July 2023 | 14 | 3 | 5 | 0 | 9 |
| 8 | September 2023 | 12 | 1 | 3 | 0 | 9 |
| 9 | November 2023 | 9 | 2 | 3 | 0 | 6 |
| 10 | February 2024 | 15 | 4 | 5 | 0 | 10 |
| 11 | April 2024 | 14 | 3 | 4 | 1 | 9 |
| 12 | July 2024 | 11 | 1 | 3 | 2 | 6 |
| 13 | October 2024 | 11 | 2 | 3 | 2 | 6 |
| 14 | January 2025 | 17 | 2 | 6 | 3 | 8 |
| 15 | April 2025 | 14 | 1 | 5 | 1 | 8 |
| 16 | June 2025 | 16 | 2 | 6 | 2 | 8 |
| 17 | October 2025 | 11 | 1 | 5 | 1 | 5 |
| 18 | December 2025 (online) | 8 | 1 | 4 | 4 | 0 |
| 19 | February 2026 | 8 | 0 | 3 | 1 | 4 |
| 20 | April 2026 | 14 | 1 | 5 | 3 | 6 |
| 21 | June 2026 (online) | 13 | 4 | 4 | 4 | 5 |
| 22 | July 2026 | 10 | 0 | 4 | 0 | 6 |
