# Supplementary material for: “It gives me confidence and value”: evaluating All_Ears@UoS patient and public involvement and engagement (PPIE) group three years on
Source: Res Involv Engagem. 2026 Oct 8;12:165. doi: 10.1186/s40900-026-00986-2 (PMC13647862; doi:10.1186/s40900-026-00986-2)
Supplement: Supplementary file 4 [file 40900_2026_986_MOESM4_ESM.docx]

**Additional file 4.** A map showing the distance in miles from University of Southampton to the surrounding areas where group member respondents live. The grey area represents the city of Southampton where the majority of respondents live.
