# Supplementary material for: “It gives me confidence and value”: evaluating All_Ears@UoS patient and public involvement and engagement (PPIE) group three years on
Source: Res Involv Engagem. 2026 Oct 8;12:165. doi: 10.1186/s40900-026-00986-2 (PMC13647862; doi:10.1186/s40900-026-00986-2)
Supplement: Supplementary file 5 [file 40900_2026_986_MOESM5_ESM.docx]

### **Additional file 5a.** Responses from rating scale questions in the group members’ survey for the statements which align with the UK Standards for Public Involvement – Communications, Governance and Impact. The number beside each statement aligns with the numbering used in figure 2 [main text].

### **Additional file 5b.** Responses from rating scale questions in the group members’ survey for the statements which align with the UK Standards for Public Involvement - Working together, Support and learning, Inclusive opportunities. The number beside each statement aligns with the numbering used in figure 2 [main text].

**Additional file 5c.** Responses from rating scale questions in the researchers’ survey for the statements which align with the UK Standards for Public Involvement – Communications, Governance and Impact. The number beside each statement aligns with the numbering used in figure 6 [main text].

**Additional file 5d.** Responses from rating scale questions in the researchers’ survey for the statements which align with the UK Standards for Public Involvement - Working together, Support and learning, Inclusive opportunities. The number beside each statement aligns with the numbering used in figure 6 [main text].
