# Supplementary material for: “It gives me confidence and value”: evaluating All_Ears@UoS patient and public involvement and engagement (PPIE) group three years on
Source: Res Involv Engagem. 2026 Oct 8;12:165. doi: 10.1186/s40900-026-00986-2 (PMC13647862; doi:10.1186/s40900-026-00986-2)
Supplement: Supplementary file 6 [file 40900_2026_986_MOESM6_ESM.docx]

**Additional file 6a.** Direct quotes from free-text responses from the group members’ survey that align with the themes and subthemes.

| **Theme or subtheme** | **Subtheme** | **Quote** | **Participant** |
| --- | --- | --- | --- |
| 1 Supporting research |  | “‘feedback on some proposed projects [G3]”. | G3 |
| 1 Supporting research |  | “‘responded to a proposed ethics document re a piece of research [G5]”. | G5 |
| 1 Supporting research |  | “articles – BMJ, CIICA and All Ears website [G15]”. | G15 |
| 1 Supporting research |  | “Helping out at open days and New Forest Shows [G11]”. | G11 |
| 1 Supporting research |  | “Networking, volunteering, identifying additional cross over opportunities with work and mentoring. Helping researchers with their project [G10]”. | G10 |
| 1 Supporting research |  | “I've been involved in a number of research projects and articles over the last year or so and can see the impact that my contribution and the contributions of other All Ears members has had [G15]”. | G15 |
| 1 Supporting research |  | “Great opportunity to meet up with others within the hearing loss community, and to be able to contribute to research and development to help others like us..[G14]”. | G14 |
| 1 Supporting research |  | “From acquiring data from those who are living daily with the challenges that hearing loss brings, develop more targeted/specific research programs designed to help support/alleviate or overcome these challenges [G6]”. | G6 |
| 1 Supporting research |  | “Being part of a group of people who understand the daily struggles hearing loss brings and knowing that contributing my thoughts and experiences of how hearing loss affects me, is valued and used towards designing future research and awareness programmes [G6]”. | G6 |
| 2 Learning opportunities and knowledge exchange | 2.1 Knowledge exchange (outward facing) | “The ALL-EARS group have learnt much from each other [G1]”. | G1 |
| 2 Learning opportunities and knowledge exchange | 2.1 Knowledge exchange (outward facing) | “‘spreading tips about how to make life easier, e.g. Speech to Test apps [G11]”. | G11 |
| 2 Learning opportunities and knowledge exchange | 2.1 Knowledge exchange (outward facing) | “Everyone listens to each other [G17]”. | G17 |
| 2 Learning opportunities and knowledge exchange | 2.1 Knowledge exchange (outward facing) | “Personal benefit from listening to sharing by researchers and others [G5]”. | G5 |
| 2 Learning opportunities and knowledge exchange | 2.1 Knowledge exchange (outward facing) | “Since attending the group I have learned so much. Each meeting there is something new to learn and find out more about [G17]”. | G17 |
| 2 Learning opportunities and knowledge exchange | 2.2 Personal learning (inward facing) | “I don't recall learning opportunities as being part of the ToR for group members, but there will certainly be opportunities for researchers and staff [G10]”. | G10 |
| 2 Learning opportunities and knowledge exchange | 2.2 Personal learning (inward facing) | “There are many reasons I enjoy being a member of ALL_EARS is because I can learn new skills and learn new things and use my life experience positively [G8]”. | G8 |
| 2 Learning opportunities and knowledge exchange | 2.2 Personal learning (inward facing) | “Also interesting to see how complex the research process is and understand how various departments and organisations work together [G15]”. | G15 |
| 3 Raising awareness | 3.1 Raising awareness within deaf and hard of hearing community | “Helping the deaf community learn about new advancements [G4]” | G4 |
| 3 Raising awareness | 3.1 Raising awareness within deaf and hard of hearing community | “Raising awareness in the local and wider community of the help and support that is available for those with deafness [G6]”. | G6 |
| 3 Raising awareness | 3.2 Raising awareness and sharing knowledge with the wider community | “I spread the word in the community. There are a lot of people with hearing loss and they don’t bother go for diagnosis especially ethnic minorities [G2]”. | G2 |
| 3 Raising awareness | 3.2 Raising awareness and sharing knowledge with the wider community | “The most important thing is the awareness as a group we are able to share with particularly the grassroot community [G9]”. | G9 |
| 3 Raising awareness | 3.2 Raising awareness and sharing knowledge with the wider community | “educating the community [G16]”. | G16 |
| 3 Raising awareness | 3.2 Raising awareness and sharing knowledge with the wider community | “I feel this project is for all communities. We run a community group, and I invited the staff to come and share contents of this project with our group. I was delighted when they said yes. Also, from that I managed to recruit a member with severe hearing loss, and she now comes to the meeting [G2]”. | G2 |
| 3 Raising awareness | 3.2 Raising awareness and sharing knowledge with the wider community | “From an intergenerational viewpoint, building knowledge within the schools as a means to help the family, would be really helpful [G1]”. | G1 |
| 3 Raising awareness | 3.2 Raising awareness and sharing knowledge with the wider community | “Also, reaching out to healthcare facilities on ways in which ALL_EARS could better serve the community [G1]”. | G1 |
| 3 Raising awareness | 3.3 Using public engagement activities to raise awareness | “Have only been able to attend a few meetings but have taken part in a few shows and exhibitions to raise awareness of cochlear implants and importance of hearing health [G13]”. | G13 |
| 3 Raising awareness | 3.3 Using public engagement activities to raise awareness | “activities to raise awareness of effects of deafness to general public [G13]”. | G13 |
| 3 Raising awareness | 3.3 Using public engagement activities to raise awareness | “In terms of wider society, the hearing tests we did at the Science Festival did a great job in raising awareness of hearing loss and maybe helped to reduce the stigma in seeking help at an early point [G15]”. | G15 |
| 3 Raising awareness | 3.3 Using public engagement activities to raise awareness | “At public events such as the Science and Engineering Festival more spots to record interactions would be helpful as neither ‘staff’ or those who participate in activities can always reach the relevant spot. A book for the public to raise issues would be Helpful [G8]”. | G8 |
| 3 Raising awareness | 3.3 Using public engagement activities to raise awareness | “listening breaks for all on the stand at Open Days as prolonged exposure to noise is harmful [G8]”. | G8 |
| 4 Impact, benefits and opportunities the group brings | 4.1 Impact of the group | “better awareness of deaf challenges [G16]”. | G16 |
| 4 Impact, benefits and opportunities the group brings | 4.1 Impact of the group | “greater awareness of the impact of deafness on the individual which means I now communicate differently. I'm more aware of how deafness can affect the lives of all involved not just the person who is hard of hearing [G14]”. | G14 |
| 4 Impact, benefits and opportunities the group brings | 4.1 Impact of the group | “My impact was clearly shown on the one aspect I've given feedback so far [G5]”. | G5 |
| 4 Impact, benefits and opportunities the group brings | 4.1 Impact of the group | “I've been involved in a number of research projects and articles over the last year or so and can see the impact that my contribution and the contributions of other All Ears members has had [G15]”. | G15 |
| 4 Impact, benefits and opportunities the group brings | 4.1 Impact of the group | “In the early formation of the group, I thought we were having a strong impact. I believe this group helps the researchers with a source of information but haven’t seen any output to show how effective the group has been for the money invested [G10]”. | G10 |
| 4 Impact, benefits and opportunities the group brings | 4.1 Impact of the group | “As we as a group moves forward it will be interesting to see how ALL-Ears and our participation impacts Hearing Loss and Cochlear Implant across the Nation/local environment [G1]”. | G1 |
| 4 Impact, benefits and opportunities the group brings | 4.2 Personal benefit of being involved | “It gives me confidence and value. When working as a deaf person these were constantly undermined. The more I learn the more useful I feel as I walk alongside prospective implantees [G8]”. | G8 |
| 4 Impact, benefits and opportunities the group brings | 4.2 Personal benefit of being involved | “Keeping up to date with innovations and research, opportunity to share experiences with the community, confidence in my own ability as a CI user to take part in group meetings, an opportunity to give and show appreciation of how my CI has changed my world.. [G13]”. | G13 |
| 4 Impact, benefits and opportunities the group brings | 4.2 Personal benefit of being involved | “There is a feeling during sessions that the individual group members contribution is valued [G1]”. | G1 |
| 4 Impact, benefits and opportunities the group brings | 4.2 Personal benefit of being involved | “Being part of a group of people who understand the daily struggles hearing loss brings and knowing that contributing my thoughts and experiences of how hearing loss affects me, is valued and used towards designing future research and awareness programmes [G6]”. | G6 |
| 4 Impact, benefits and opportunities the group brings | 4.2 Personal benefit of being involved | “Personally, I've appreciated being asked for my opinion and being given the opportunity to share my experiences or help to make a research study more inclusive or relevant for other patients like me. The researchers I've been involved with have genuinely appreciated my comments [G15]”. | G15 |
| 4 Impact, benefits and opportunities the group brings | 4.2 Personal benefit of being involved | “Great opportunity to meet up with others within the hearing loss community [G15]”. | G15 |
| 4 Impact, benefits and opportunities the group brings | 4.2 Personal benefit of being involved | “Get to keep up to date with the latest research and able to contribute [G3]”. | G3 |
| 4 Impact, benefits and opportunities the group brings | 4.2 Personal benefit of being involved | “Keeping up to date with innovations and research [G13]”. | G13 |
| 4 Impact, benefits and opportunities the group brings | 4.3 Opportunities the group brings | “lots of opportunities over the last year to be involved in a diverse range of projects [G15]”. | G15 |
| 4 Impact, benefits and opportunities the group brings | 4.3 Opportunities the group brings | “giving something back [G18]”. | G18 |
| 4 Impact, benefits and opportunities the group brings | 4.3 Opportunities the group brings | “a chance to eventually have an impact on the lives of people like me [G5]”. | G5 |
| 4 Impact, benefits and opportunities the group brings | 4.3 Opportunities the group brings | “Being a member of a group who has the ability to impact and benefit our community. Often, you really do not know what you know until you have a conversation with someone and realise that you are in a position to give them even a small direction on how to seek help. We do not know so much but the little we know, can really make a change in someone's day [G1]”. | G1 |
| 5 Diversity and inclusivity within the group | 5.1 Involvement with diverse communities | “the group is diverse [G2]”. | G2 |
| 5 Diversity and inclusivity within the group | 5.1 Involvement with diverse communities | “engaging everyone from various backgrounds [G3]”. | G3 |
| 5 Diversity and inclusivity within the group | 5.1 Involvement with diverse communities | “‘integration and inclusion of the multicultural diverse community [G9]”. | G9 |
| 5 Diversity and inclusivity within the group | 5.1 Involvement with diverse communities | “On the grounds that there is always room for improvement there could be more diversity but any lack is due more to people not coming forward rather than lack of opportunity to do so. The All_Ears organisers try hard and in inventive ways to approach as wide a range of age, ethnicity, gender, and type of hearing loss as possible [G8]”. | G8 |
| 5 Diversity and inclusivity within the group | 5.1 Involvement with diverse communities | “encouragement of a wide range of participants with lived experience of varying degrees of hearing loss [G6]”. | G6 |
| 5 Diversity and inclusivity within the group | 5.2 The group is inclusive | “We are all treated equally [G2]”. | G2 |
| 5 Diversity and inclusivity within the group | 5.2 The group is inclusive | “The meeting is inclusive and I never feel out of place [G2]”. | G2 |
| 5 Diversity and inclusivity within the group | 5.2 The group is inclusive | “very inclusive and accessible meetings [G2]”. | G2 |
| 5 Diversity and inclusivity within the group | 5.2 The group is inclusive | “attention to accessibility in the group meeting [G5]”. | G5 |
| 5 Diversity and inclusivity within the group | 5.2 The group is inclusive | “The meetings are transcribed so that we can read what is being said during presentations [G17]”. | G17 |
| 5 Diversity and inclusivity within the group | 5.2 The group is inclusive | “My response focuses on the single approach for holding meetings in person. For those who work, is the meeting time/place inclusive or simply ensuring the meeting time is conducive for staff and researchers but not necessarily for members [G10]”. | G10 |
| 5 Diversity and inclusivity within the group | 5.3 Lack of involvement from some communities | “I'm not sure the processes that are followed ensure the group is inclusive and diverse and alongside Q39, what baseline is being used to gauge whether you have true representation. The question says Deaf (capital D) or hard of hearing but is it in fact a group of deaf (lower case d) and hard of hearing people with lived experience rather than people from the Deaf Community who do not generally advocate for cochlear implants [G9]”. | G9 |
| 5 Diversity and inclusivity within the group | 5.3 Lack of involvement from some communities | “I do not recall any members who were born deaf and not subsequently fitted with CI's [G11]”. | G11 |
| 5 Diversity and inclusivity within the group | 5.3 Lack of involvement from some communities | “Less young people in their 20s as members are involved, probably because of work commitments [G7]”. | G7 |
| 5 Diversity and inclusivity within the group | 5.3 Lack of involvement from some communities | “We don't have any BSL users in the group. We don't have many younger members, although I know this is being partially addressed by the young people's group that is being established [G15]”. | G15 |
| 5 Diversity and inclusivity within the group | 5.3 Lack of involvement from some communities | “being optimistic about the commitment of the group for teens [G17]”. | G17 |
| 6 Running the group | 6.1 Leadership of the group | “To some degree leadership (provided by Kate) was necessary for most of the questions above (regarding the ‘Working together’ statements for the rating scale questions). Without her tactful leadership the group would not have been formed nor survived [G11]”. | G11 |
| 6 Running the group | 6.1 Leadership of the group | “The university team is very enthusiastic in what they do, and this really captures our attention [G2]”. | G2 |
| 6 Running the group | 6.1 Leadership of the group | “directive from senior academics for meaningful PPI and a dedicated researcher working with the representatives [G12]”. | G12 |
| 6 Running the group | 6.1 Leadership of the group | “UoS resources rely mainly on Kate. Some diversification would be useful in case she becomes unavailable [G11]”. | G11 |
| 6 Running the group | 6.1 Leadership of the group | “regular written communication, before, during and after every meeting which is clear to all [G6]”. | G6 |
| 6 Running the group | 6.1 Leadership of the group | “I do not know if anyone who would like to receive All_Ears communication by means other than email is able to or is deterred from joining because they do not have email or an email ‘ buddy’ to keep them in the loop. For myself I am very pleased with the level and clarity of email communication. Emails I have sent always have a prompt response as well [G8]”. | G8 |
| 6 Running the group | 6.1 Leadership of the group | “Not all members access email. However, the details (date/time/location) of the next meeting is shared at the end of each meeting [G1]”. | G1 |
| 6 Running the group | 6.2 Areas that are working well | “The environment and group leaders are very welcoming. The group is friendly and everyone listens to each other [G17]”. | G17 |
| 6 Running the group | 6.2 Areas that are working well | “mutual support [G11]”. | G11 |
| 6 Running the group | 6.2 Areas that are working well | “Being part of a friendly, like-minded group of people with shared lives experiences [G18]”. | G18 |
| 6 Running the group | 6.2 Areas that are working well | “friendly, helpful group [G4]”. | G4 |
| 6 Running the group | 6.2 Areas that are working well | “My first reaction to this question is not to try to fix something already working well [G11]”. | G11 |
| 6 Running the group | 6.2 Areas that are working well | “relaxed setting where people can interact both sat round table formally and less formal too in coffee break too [G7]”. | G7 |
| 6 Running the group | 6.2 Areas that are working well | “To be honest, at the moment we are doing great! Well done to the team [G17]”. | G17 |
| 6 Running the group | 6.2 Areas that are working well | “It is wonderful meeting with the members rather than having online participation [G1]”. | G1 |
| 6 Running the group | 6.2 Areas that are working well | “Joining together with others, those that have hearing loss and those that work in community [G7]”. | G7 |
| 6 Running the group | 6.3 Areas that need improvement | “The reimbursement of participants can take several weeks. This needs to improve especially being such a credible University. Finance teams are slow or don’t value public contributors [G2]”. | G2 |
| 6 Running the group | 6.3 Areas that need improvement | “Like I have said all along, your finance team may put off participation. The don’t seem to understand how to value the public. It is a university after all. Clever people! [G2]”. | G2 |
| 6 Running the group | 6.3 Areas that need improvement | “Barriers to the reimbursement process have been identified and, in some cases, still needs to be looked into [G1]”. | G1 |
| 6 Running the group | 6.3 Areas that need improvement | “My only comment is regarding finance team at the University. They don't seem to value public involvement. One member does not come any more due to this [G2]”. | G2 |
| 6 Running the group | 6.3 Areas that need improvement | “Not all members access email [G1]”. | G1 |
| 6 Running the group | 6.3 Areas that need improvement | “I do not know if anyone who would like to receive All_Ears communication by means other than email is able to or is deterred from joining because they do not have email or an email ‘ buddy’ to keep them in the loop [G8]”. | G8 |
| 6 Running the group | 6.3 Areas that need improvement | “Some topics could do with longer discussion time if people are passionate about them [G16]”. | G16 |
| 6 Running the group | 6.4 Suggestions to improve meetings | “more flexibility in the times of meetings [G3]”. | G3 |
| 6 Running the group | 6.4 Suggestions to improve meetings | “if employed and working the time of the meetings make it difficult to participate as much as one would like [G3]”. | G3 |
| 6 Running the group | 6.4 Suggestions to improve meetings | “maybe online meetings for wider reach [G18]”. | G18 |

**Additional file 6b.** Direct quotes from free-text responses from the researchers’ survey that align with the themes and subthemes.

| **Theme** | **Subtheme** | **Quote** | **Participant** |
| --- | --- | --- | --- |
| 1 Benefits of working with the group for researchers | 1.1 Increased knowledge, awareness and skills | “as a researcher and clinician, it is vital for me to be able to learn from a diverse group of people with lived experience of hearing loss [R7]”. | R7 |
| 1 Benefits of working with the group for researchers | 1.1 Increased knowledge, awareness and skills | “The group has had invaluable contributions to my research including allowing me to gain more of an insight into hearing loss and helping to design studies that is most likely to benefit the people who it most affects [R4]”. | R4 |
| 1 Benefits of working with the group for researchers | 1.1 Increased knowledge, awareness and skills | “As someone newer to research, it developed my knowledge and insight into PPIE and why it is important [R4]”. | R4 |
| 1 Benefits of working with the group for researchers | 1.2 Value of working with the group members | “Access to a diverse PPIE group which is hard to find elsewhere [R6]”. | R6 |
| 1 Benefits of working with the group for researchers | 1.2 Value of working with the group members | “The opportunity to work with an interested and valuable population [R5]”. | R5 |
| 1 Benefits of working with the group for researchers | 1.3 Increased positive feeling from working with the group | “Contributed to making research more inclusive and impactful [R1]”. | R1 |
| 1 Benefits of working with the group for researchers | 1.3 Increased positive feeling from working with the group | “Boosted my motivation and sense of purpose in my research [R1]”. | R1 |
| 1 Benefits of working with the group for researchers | 1.3 Increased positive feeling from working with the group | “Increased my confidence in engaging the public meaningfully [R1]”. | R1 |
| 2 Positive impact on research and society | 2.1 Public involvement is an invaluable part of research | “Recognition of public involvement as a valuable part of the research process [R1] | R1 |
| 2 Positive impact on research and society | 2.1 Public involvement is an invaluable part of research | “The group has had invaluable contributions to my research including allowing me to gain more of an insight into hearing loss and helping to design studies that is most likely to benefit the people who it most affects [R4]”. | R4 |
| 2 Positive impact on research and society | 2.1 Public involvement is an invaluable part of research | “‘an invaluable part of research [R4]”. | R4 |
| 2 Positive impact on research and society | 2.2 Improved study design, increased relevance and real-world applicability of research | “I have been able to share my ideas and get feedback [R5]”. | R5 |
| 2 Positive impact on research and society | 2.2 Improved study design, increased relevance and real-world applicability of research | “Better-designed studies with real-world applicability [R1]’. | R1 |
| 2 Positive impact on research and society | 2.2 Improved study design, increased relevance and real-world applicability of research | “Improved research relevance and sensitivity to lived experiences [R1]”. | R1 |
| 2 Positive impact on research and society | 2.2 Improved study design, increased relevance and real-world applicability of research | “It means that research becomes relevant and focused on the users of CI and hearing technology. This has impact on national research and also society [R2]”. | R2 |
| 2 Positive impact on research and society | 2.3 Societal impact | “The group has gone from strength to strength, and this has increased representation within the group and reach into the community [R8]”. | R8 |
| 2 Positive impact on research and society | 2.3 Societal impact | “Opportunities to influence real-world change [R1]”. | R1 |
| 2 Positive impact on research and society | 2.3 Societal impact | “It means that research becomes relevant and focussed on the users of CI and hearing technology. This has impact on national research and also society [R1]”. | R1 |
| 2 Positive impact on research and society | 2.3 Societal impact | “The group has gone from strength to strength, and this has increased representation within the group and reach into the community [R8]”. | R8 |
| 3 Value of group members | 3.1 Valuable contributions from group members | “Gained insight into lived experiences that shape better research [R1]”. | R1 |
| 3 Value of group members | 3.1 Valuable contributions from group members | “It is invaluable to have insights and guidance of CI users and those with hearing loss [R2]”. | R2 |
| 3 Value of group members | 3.1 Valuable contributions from group members | “Specific, local and timely information/views from people with relevant experience [R3]”. | R3 |
| 3 Value of group members | 3.2 Positive attributes of group members | “Probably the main success of this group is they are very engaged [R7]”. | R7 |
| 3 Value of group members | 3.2 Positive attributes of group members | “This is an exceptional group! I very much value cochlear implant users have a voice in research [R2]”. | R2 |
| 3 Value of group members | 3.2 Positive attributes of group members | “It is a brilliantly diverse, skilled group of individuals [R8]”. | R8 |
| 4 Organisation and running of the group | 4.1 Areas that are working well | “It is a diverse group that contributes to research at all stages [R4]”. | R4 |
| 4 Organisation and running of the group | 4.1 Areas that are working well | “I think what is working well as far as I know is that the group is diverse [R7]”. | R7 |
| 4 Organisation and running of the group | 4.1 Areas that are working well | “The group has gone from strength to strength and this has increased representation within the group and reach into the community [R8]”. | R8 |
| 4 Organisation and running of the group | 4.1 Areas that are working well | “Clear communication and coordination from the group facilitators [R1]”. | R1 |
| 4 Organisation and running of the group | 4.1 Areas that are working well | “Good communication between researcher and (the) group [R5]”. | R5 |
| 4 Organisation and running of the group | 4.1 Areas that are working well | “Collaborative spirit and willingness from researchers to genuinely listen [R1]”. | R1 |
| 4. Organisation and running of the group | 4.1 Areas that are working well | “Probably the main success of this group is the very engaged and approachable and committed lead – Kate [R7]”. | R7 |
| 4 Organisation and running of the group | 4.1 Areas that are working well | “Fixed scheduling for meetings - our project was not discussed in person due to running out of time [R6]”. | R6 |
| 4 Organisation and running of the group | 4.2 Areas for improvement | “More frequent meetings [R2]”. | R2 |
| 4 Organisation and running of the group | 4.2 Areas for improvement | “Funding are always the issues - this is such an amazing group to work with but sometimes it would be great to have longer with them [R8]”. | R8 |
| 4 Organisation and running of the group | 4.2 Areas for improvement | “More structured follow-up to show how public feedback has influenced research [R1]”. | R1 |
