# Supplementary material for: “It gives me confidence and value”: evaluating All_Ears@UoS patient and public involvement and engagement (PPIE) group three years on
Source: Res Involv Engagem. 2026 Oct 8;12:165. doi: 10.1186/s40900-026-00986-2 (PMC13647862; doi:10.1186/s40900-026-00986-2)
Supplement: Supplementary file 7 [file 40900_2026_986_MOESM7_ESM.docx]

**Additional file 7.** Gender of researcher respondents and the institution and faculty the researchers belong to.

| **Gender** | **Number** | **Percentage of researcher respondents (%)** |
| --- | --- | --- |
| Female | 7 | 88 |
| Male | 1 | 12 |
| **Institution** |  |  |
| University of Southampton | 7 | 88 |
| Univerisity of Cambridge | 1 | 12 |
| **Faculty** |  |  |
| Social Sciences | 1 | 12 |
| Faculty of Engineering and Physical Science | 1 | 12 |
| Faculty of Enviornmental and Life Sciences | 1 | 12 |
| Faculty of Medicine | 2 | 24 |
| Clinical Neurosciences | 1 | 12 |
| Auditory Implant Service | 1 | 12 |
| **Current position** |  |  |
| Postgraduate researcher | 0 | 0 |
| Postdoctoral researcher | 0 | 0 |
| Academic | 1 | 12 |
| Clinician | 2 | 24 |
| Academic and clinician | 3 | 36 |
| Undergraduate | 1 | 12 |
| Project manager | 1 | 12 |
